# Automated insect detection and biomass monitoring via AI and electrical field sensor technology

**DOI:** 10.1038/s41598-025-15613-5

**Published:** 2025-08-14

**Authors:** Freja Balmer Odgaard, Páll Vang Kjærbo, Amir Hossein Poorjam, Khaled Hechmi, Rubens Monteiro Luciano, Niels Krebs

**Affiliations:** FaunaPhotonics, Oceanvej 1, 2150 Copenhagen, Denmark

**Keywords:** Insects, Biomass, Insect monitoring, Signal processing, Artificial intelligence, Convolutional neural network, Wing beat frequency, Electrical and electronic engineering, Entomology, Machine learning

## Abstract

Insects, vital for ecosystem stability, are declining globally necessitating improved monitoring methods. Trap-based approaches are labor-intensive, invasive, and limited in scope. This study therefore presents a novel, automated, non-invasive insect monitoring system that detects atmospheric electrical field modulations caused by flying insects. In-field sensors monitor insect activity and biomass without physical trapping, using differential electric field measurements and convolutional neural networks for detection and wing-beat frequency analysis. Furthermore, a biomass algorithm that estimates taxon-specific weights is introduced. To validate this method, paired sensor and Townes Malaise trap deployments were conducted at two sites in a Danish nature reserve. Results showed moderate to strong correlations between sensors and traps, particularly at one site (Spearman’s $\rho =0.725$ for counts; 0.644 for biomass), supporting the method’s viability. A discrepancy in biomass estimates between methods, greater than that of counts, suggests the need for further refinement of the sensor’s biomass estimation. For inter-method consistency, sensor-sensor correlations ($\rho =0.758$ for counts; 0.867 for biomass) exceeded Malaise-Malaise correlations ($\rho =0.597$ for counts; 0.641 for biomass), though not significantly so ($P=0.304$ for counts; $P=0.057$ for biomass). Overall, the study concludes that while further work is needed, this innovative approach shows promise for future insect monitoring and ecological research.

## Introduction

Insects, which comprise over half of all described species^1^, perform crucial functions that keep our ecosystems balanced, including pollination, nutrient cycling, and pest control^2–5^. In recent years, however, numerous studies have reported alarming insect declines in abundance, biomass, and species richness^6–10^, with human-driven habitat change being the greatest driver^11,12^. Monitoring insects is therefore essential to ensure proper conservation^13^ and safeguard the ecosystem services they provide^2,14^. Insects also function as indicators of environmental health and overall biodiversity^13,15,16^, further cementing the need for capable insect monitoring methods.

Entomologists use a variety of insect monitoring techniques, including pan-, pit-, light-, and Malaise-traps^17,18^. However, these require high levels of labor, both during collection of insects and later in sorting, counting, and weighing^18,19^. These methods also remove insects from the population, potentially affecting local population dynamics and threatening rare or fragile species^20^. The extent of this impact remains unclear, raising ethical concerns, especially as the use of lethal sampling methods continues to increase despite growing conservation awareness^20,21^.

Automating the monitoring process would solve the issue of labor intensity, and for non-lethal methods, also eliminate the ethical concerns associated with lethal sampling. Additionally, automation would improve the granularity of temporal data and facilitate simultaneous monitoring across large spatial areas. A review by van Klink et al.^22^ outlines the rapidly evolving field of automated insect monitoring methods and discusses their potential and current limitations. Examples of such automated methods include LiDAR^23^ and computer vision^24^.

To address this need for automated monitoring, we present a novel method utilizing electrical field sensors for passive and non-invasive insect monitoring. The sensor detects flying insects by analyzing disruptions in the atmospheric electric field caused by insect flight in combination with the triboelectric charge accumulation of the insect. Using a differential probe design, the sensor enhances signal detection while suppressing environmental noise. Signals are then preprocessed to remove power line interference, enhancing the signal-to-noise ratio. A convolutional neural network (CNN) then detects flying insects in the signal by classifying segments based on presence. For segments containing insect activity, wing-beat frequencies (WBFs) are estimated using the probabilistic YIN algorithm^25^. WBFs are then used to estimate biomass based on a reference table that maps WBFs to body mass for a wide range of insect taxa. Furthermore, to validate this method in the field, we statistically evaluated sensor estimates against data collected from the widely used Townes Malaise trap^18,26^.

## System overview

**Fig. 1:**
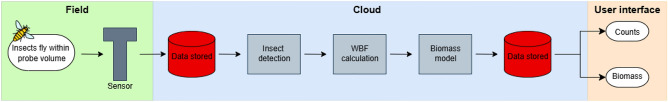
System block diagram. From left to right: In-field sensor transmits electrical field data to the cloud. Cloud-hosted AI interpretation of data to extract insect events, calculate WBF, and estimate biomass. Via a user interface, count and biomass values are available.

The complete system architecture comprises three main components: field sensor units, cloud processing infrastructure, and a user interface, as illustrated in Fig. 1. At the core of the field deployment is the electrical field sensor (hereafter referred to as sensor), which utilizes an electrical field detection principle coupled with an analog-to-digital converter and a microcontroller for signal acquisition and transmission. Field data is continuously transmitted to a cloud-based processing pipeline. The cloud processing system prepares the received signals by removing power line noise, detects the presence of flying insects, calculates WBF, and estimates the body mass. The processed data, aggregated to estimate biomass and activity, is made accessible through a user interface for detailed analysis and data export. This integrated approach enables continuous, automated monitoring of flying insects in the monitored area.

## Operating principle of the sensor

The sensor employed in this study exploits natural electrical effects. As insects fly, they generate unique electrical signatures through two mechanisms: by disrupting the ambient field between the atmosphere and the Earth’s surface and by acquiring a positive charge through triboelectric effects from air friction^27^. In fact, the importance of triboelectricification in insect ecology is an emerging topic^27^, with many studies revealing how insects utilize this phenomenon. A study on bumblebees (*Bombus terrestris*) demonstrates that this positive charge aids in pollen-gathering behavior since flowers tend to be negatively charged^28^. Additionally, honeybees (*Apis mellifera*) have been shown to use electric field charges in social communication^29^. This positive charge acquisition during flight, combined with the field disruption, creates distinctive electrical signatures that can be detected and analyzed.

The concept of using an electric field antenna to detect flying insects was demonstrated by Koemel and Callahan^30^. In their study, insects were placed in a 23 cm high screen-wire cylinder with a 13 cm diameter and “stimulated to fly”, while a radio receiver coupled to the electric field antenna recorded the signals. The screen-wire cylinder acted as both an insect cage and a Faraday cage, minimizing electric noise and ensuring accurate signal capture. However, this approach is unsuitable for in-field measurements as it requires a controlled Faraday cage environment.

To enable field measurements, our sensor system employs a differential measurement approach using two identical electrostatic probes. This design effectively mitigates the influence of distant noise sources such as atmospheric disturbances, radio signals, and power line interference, as these produce common-mode signals of equal amplitude at both probes. The differential approach enhances the sensor’s ability to detect nearby insects while suppressing environmental far-field noise, quantified by the sensor’s common-mode rejection ratio. However, power line noise from nearby sources presents a challenge to common mode rejection due to their physical length and varying distances.

**Fig. 2:**
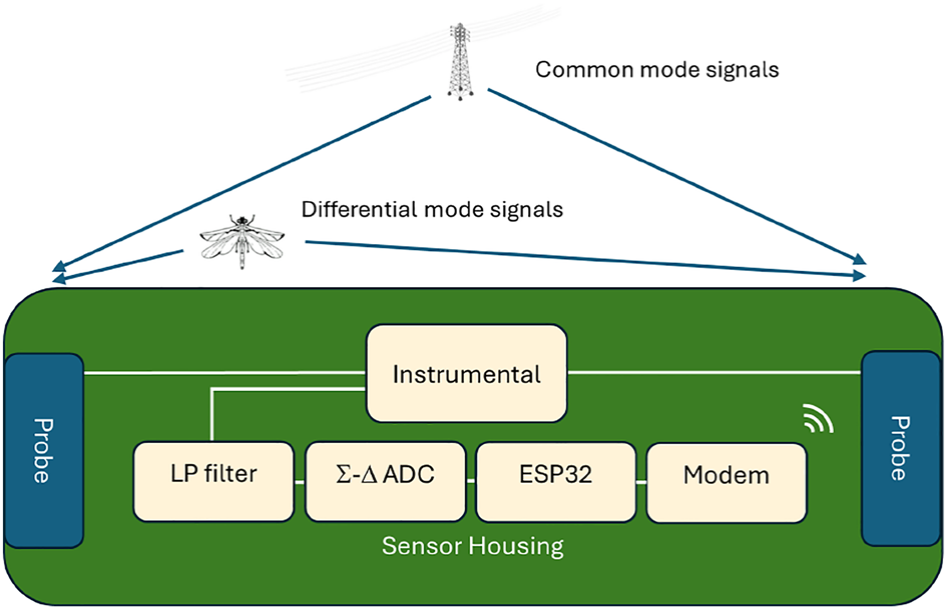
Schematic overview of the electric field sensor architecture. Signals from the two identical electrostatic probes are inputs to the instrumental amplifier, amplifying differential signals while suppressing common-mode signals. The low-pass (LP) filter further suppresses unwanted signals before digitization by the $\Sigma$-$\Delta$ ADC. An ESP32 micro-controller and modem handle the preprocessing of signals and subsequent upload to the cloud. Key specifications include a probe area of $75~\text {cm}^2$ per probe, a probe spacing of 28 cm, input impedance $>1$ $T\Omega$, and a sampling rate of 4 kHz.

The probes are positioned 28 cm apart within a weatherproof housing (Fig. 2). Each probe features a conductive surface area of approximately 75 cm^2^, specifically designed to optimize electrostatic field sensitivity while minimizing electromagnetic interference susceptibility. The probe geometry and spacing configuration represent a design optimization: increased separation enhances differential signal detection from nearby insects but reduces system compactness, while decreased separation improves portability at the expense of detection sensitivity. The detection of picocoulomb-level electrostatic signals necessitates specialized analog signal conditioning. The instrumentation amplifier incorporates very high input impedance ($>10^{12}$ $\Omega$) to minimize probe loading effects and preserve the weak electrostatic signatures. Bandwidth limiting through a low-pass filter attenuates radio frequency and radar interference while preserving insect WBFs (5-2,000 Hz). Digital conversion utilizes a $\Sigma$-$\Delta$ ADC architecture, selected for its inherent anti-aliasing characteristics and high-resolution capabilities. An ESP32 micro-controller performs real-time signal preprocessing and manages data transmission protocols via cellular communication to cloud-based processing infrastructure.

**Fig. 3:**
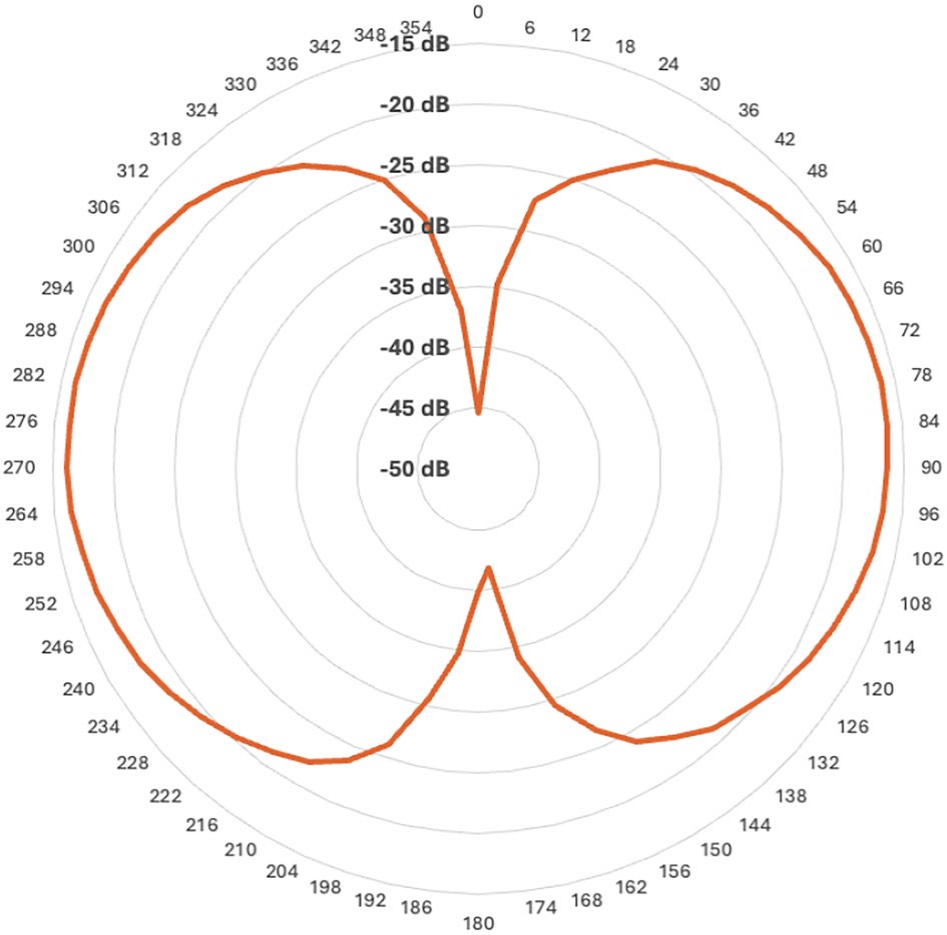
Sensitivity radar plot in dB for a sensor. The vertical symmetry plane between $0^\circ$ and $180^\circ$ corresponds to the region of lowest sensitivity due to the differential probe design.

This passive sensing method provides a novel approach to insect monitoring without requiring active signal emission. The differential sensor design creates a distinctive detection volume comprising two spherical shells with a common interface. The sensitivity distribution within this volume is characterized by maximum values directly in front of each probe, with detection distances typically exceeding 1 meter. However, the sensor’s detection capability exhibits a size-dependent sensitivity pattern, where larger insects generate proportionally stronger signals. This relationship creates an inherent bias in detection probability, particularly at the outer boundaries of the detection volume, where smaller insects may go undetected while larger ones remain detectable. A notable characteristic of the differential design is the existence of a symmetry plane between the two probes where sensitivity approaches zero, creating a “blind plane” in the detection volume. This feature, inherent to differential-based sensors, is clearly visualized at $0^\circ$ and $180^\circ$ in Fig. 3, which presents the radar plot of sensitivity measurements in decibels (dB) for a sensor.

## Data processing

**Fig. 4:**
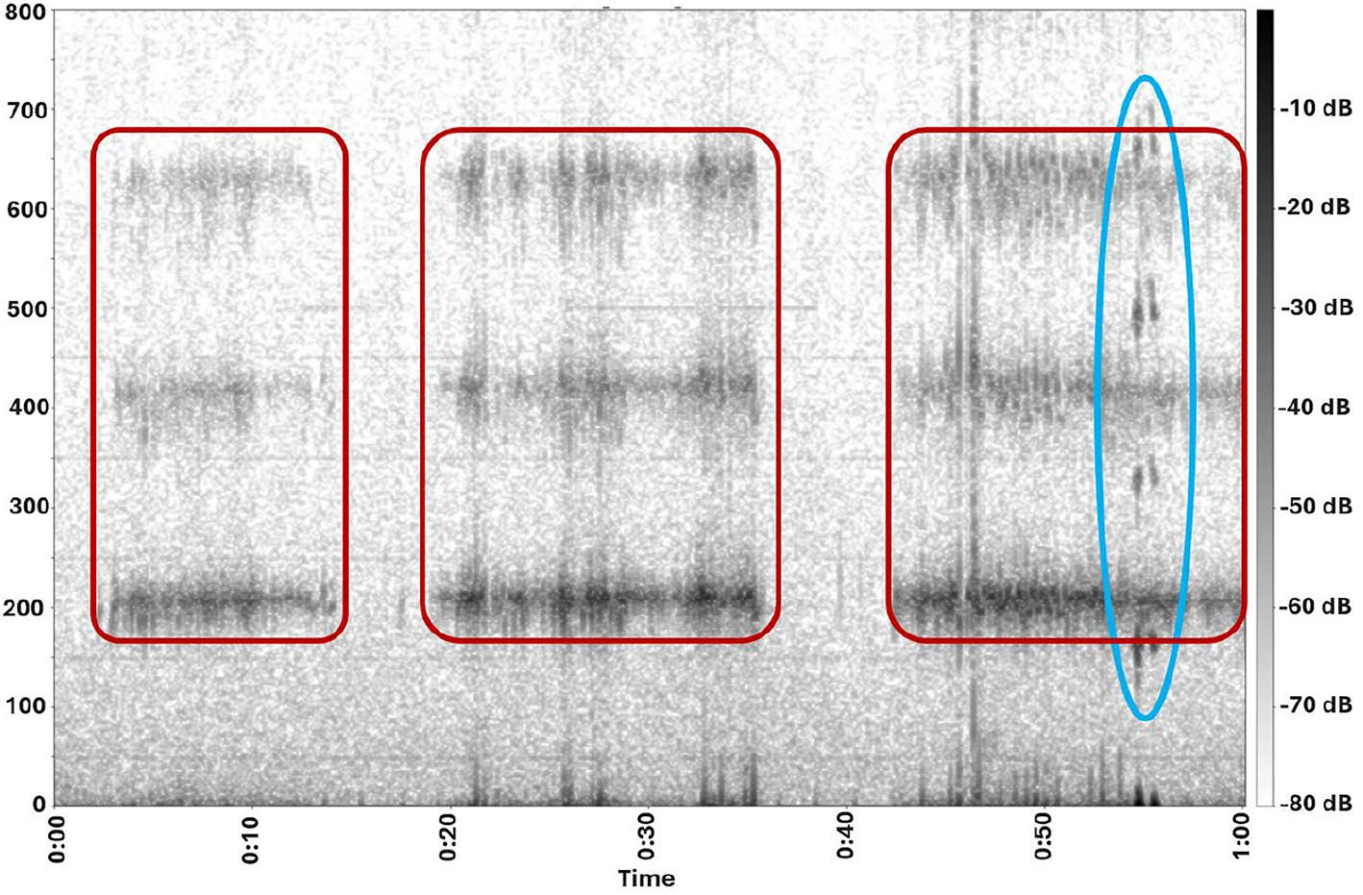
Spectrogram of a 1-minute signal showing the activities of a bumblebee (*Bombus spp.*, Apidae, Hymenoptera) (red) with a WBF of $\approx 210$ Hz, and a fly (Brachycera, Diptera) (blue) with a WBF of $\approx 160$ Hz.

In this section, we detail the methodologies employed for signal preprocessing, insect detection, and biomass calculation, as outlined in Supplementary Algorithm S1. The sensors record data in 1-minute intervals, producing 16-bit signals sampled at a frequency of 4 kHz. This sampling frequency, selected per the Nyquist criterion, ensures accurate capture of insect signal frequencies up to 2 kHz, enabling effective analysis while minimizing data size for efficient transmission and storage. Figure 4 shows the spectrogram of a sample 1-minute signal, capturing the activities of two different insects flying close to the sensor at distinct WBFs.

### Power line noise detection and cancellation

The sensors are sensitive to surrounding electrical fields, making them prone to power line interference. Power line noise refers to the electromagnetic interference caused by the alternating current power supply typically found in electrical grids. This interference occurs at specific frequencies, commonly 50 Hz and 60 Hz, depending on the geographical region. To address this, we implemented a power line noise detection algorithm that calculates the power spectral density (PSD) of the input signal using the Welch method^31^ and checks if the energy in the specified frequency bands (50 Hz and 60 Hz) is greater than in the adjacent bands. To reduce the risk of misdetection due to insect WBFs similar to the power line frequency, the signal is split into four chunks, and power line presence is confirmed only if detected in all segments. Upon detection, a comb filter—a type of notch filter designed to remove periodic noise components by applying multiple narrow-band stop filters at harmonic frequencies^32^—is applied to cancel the power line component and its harmonics.

### Insect detection

The detection of insects was achieved using a CNN optimized for classifying signals into “*insect*” or “*non-insect*” categories.

#### Signal segmentation and preprocessing

The CNN requires fixed-length inputs, so signals were divided into 1-second segments. This length captures multiple wing-beat cycles for reliable feature extraction while maintaining computational efficiency. If a segment at the end of a recording is shorter than 1 second, it is discarded.

Each segment, consisting of 4,000 samples at a 4 kHz sampling rate, was converted to a spectrogram (129$\times$32) using the short-time Fourier transform (STFT), preserving both time and frequency information for distinguishing insect events.

#### CNN architecture

The CNN processed the spectrograms through:

- **Convolutional layers**: Two convolutional layers of 16 and 32 filters. Both layers employ a rectified linear unit (ReLU) activation function and are followed by batch normalization to stabilize and accelerate the training process.
- **Max-pooling layer**: Reduces spatial dimensions to highlight key features, lowering computational load and reducing the risk of overfitting.
- **Fully connected layers**: A dense layer with 256 neurons, followed by batch normalization and ReLU activation. The final dense layer consists of a single unit, which outputs the binary classification result (insect or non-insect).
- **Dropout layers**: Applied to prevent overfitting by randomly deactivating a fraction of neurons during training, ensuring regularization across the network.

#### Training and data augmentation

The training dataset consisted of 14,481 insect segments and 96,988 non-insect segments, taken from 56 sensors. No rebalancing was done, as the loss function was weighted based on the positive/negative class ratio. To enhance model robustness and address class imbalance, several data augmentation techniques were applied to both classes, including pitch shifting of ±4 semitones, time shifting with a factor between $-0.5$ and $+0.5$ measured in fractions of the total length of the segment, and time stretching with a factor between 0.8-1.25. Noise addition was also applied, comprising both Gaussian noise (amplitude of 0.003) and real-world noise segments extracted from sensor recordings not used in training. The real-world noise segments represent signal intervals containing non-insect disturbances or interfering events that may confound insect detection. Applying augmentation to both classes prevents the model from associating augmentation artifacts with the insect class, ensuring a more balanced learning process. Augmentation was performed at each epoch with a probability of 50% on each segment. If the augmentation was performed on segment *i*, then each technique has a 50% probability of being applied to segment *i*. These augmentations simulate variability in environmental conditions and insect signals, ensuring the model generalizes well across diverse scenarios. Detected signals are processed for WBF analysis and biomass estimation.

#### Model validation

To determine the most effective CNN architecture described in the previous sections, a dataset was curated through a dedicated Streamlit-based interface. This allowed for inspection of spectrograms and for manual annotation of regions containing insect activity by drawing bounding boxes around visible signal patterns. In addition to visual inspection, the recordings were audibly reviewed to reduce the likelihood of mislabeling non-insect interferences (e.g., environmental or electrical noise) as insect events.

- For labeled segments longer than one second, the duration was rounded down to the nearest whole second to ensure each extracted segment contained a representative portion of the insect signal. This mitigates the risk of including segments dominated by silence or irrelevant signals. One-second non-overlapping segments were then extracted for model training.
- For labeled segments shorter than one second, symmetric padding with adjacent signal portions was applied to reach the required length. Padding was taken equally from before and after the annotation, unless limited by the recording boundaries. The added context was minimal and did not constitute a substantial portion of the final segment, thus preserving label integrity.

The annotated dataset was sourced from 129 distinct sensor units and was partitioned into three non-overlapping subsets to prevent data leakage and ensure proper generalization across devices and locations. Specifically, data from 56 sensors were used for training, ensuring sufficient representation of both insect and non-insect classes. A separate validation set from 39 sensors was used for model selection and hyperparameter tuning. Finally, a test set comprising data from the remaining 34 sensors was reserved exclusively for performance evaluation, simulating real-world deployment conditions by ensuring the model had no prior exposure to this subset.

Evaluation on the held-out test set, which included 4,949 insect-labeled segments and 52,760 non-insect segments, demonstrated high classification performance. The model achieved an area under the receiver operating characteristic curve (AUC) of 0.96, with an F1-score of 0.79, a precision of 0.77, and a recall of 0.81. These results indicate strong discriminative capability, balancing sensitivity and specificity in identifying insect activity across diverse recording conditions.

### WBF calculation

For WBF calculation, we employ the probabilistic YIN algorithm^25^, an extension of the YIN algorithm^33^ for fundamental frequency (F0) estimation. The pYIN algorithm first computes F0 candidates and their probabilities using the YIN algorithm, followed by Viterbi decoding to estimate the most likely F0 sequence and voicing flags. Fundamental frequencies are calculated for short frames where the signal is assumed to be stationary. For the $i^{\textrm{th}}$ 1-second segment, we compute the mean and standard deviation of the fundamental frequencies, denoted as $\mu _{f_0}^{i}$ and $\sigma _{f_0}^{i}$, respectively. To further refine the accuracy of the detection, segments with $\mu _{f_0}<20$ Hz, $\sigma _{f_0}<1$ Hz, and $\frac{\sigma _{f_0}}{\mu _{f_0}}<0.8$ are discarded. These thresholds were selected based on specific criteria and adjusted experimentally: we focused on insect wing-beats, which were generally above 20 Hz. This lower threshold filters out low-frequency noise caused mostly due to small vibrations of the sensors being exposed to wind in open fields. The criterion $\sigma _{f_0}<1$ Hz ensures that segments with very stable frequencies, typically indicative of non-insect signals, are excluded, as we assume that the WBF of a flying insect is not constant over time. The ratio of standard deviation to mean frequency, $\frac{\sigma _{f_0}}{\mu _{f_0}}$, was set to discard segments where the WBF drastically changes, which helps to eliminate noisy or unreliable segments. This post-processing step significantly reduces the false positives generated by the CNN model, ensuring that only reliable insect detections are considered for subsequent analysis.

### Insect segments aggregation

After initial insect event detection and WBF calculation, an additional post-processing step is applied to determine whether the detected insect events from adjacent segments originate from the same insect or from different insects. Adjacent segments are aggregated if the following condition are met:

1 $$\begin{aligned} IoU(i, i+1)&= \frac{|E_i \cap E_{i+1}|}{|E_i \cup E_{i+1}|} \ge 0.1 \quad&\textrm{BC}(\mu _{f_0}^{i}, \sigma _{f_0}^{i}, \mu _{f_0}^{i+1}, \sigma _{f_0}^{i+1})&\ge 0.7 \end{aligned}$$

where $E_i = [\mu _i - \frac{\sigma _{i}}{2}, \mu _i + \frac{\sigma _{i}}{2}]$ is the $i^{\textrm{th}}$ interval, and *BC* is the Bhattacharyya coefficient^34^ between the segments calculated as:

2 $$\begin{aligned} \textrm{BC}(\mu _{f_0}^{i}, \sigma _{f_0}^{i} , \mu _{f_0}^{i+1}, \sigma _{f_0}^{i+1}) = \frac{1}{4}\frac{(\mu _{f_0}^{i} - \mu _{f_0}^{i+1})^2}{{\sigma _{f_0}^{i}}^2 + {\sigma _{f_0}^{i+1}}^2}+\frac{1}{2}\ln \left( \frac{{\sigma _{f_0}^{i}}^2 + {\sigma _{f_0}^{i+1}}^2}{2\sigma _{f_0}^{i} \sigma _{f_0}^{i+1}}\right) \end{aligned}$$

This aggregation method assumes that segments meeting these criteria are likely to be generated by the same insect, spanning multiple 1-second intervals. The WBFs of these segments are then merged and the mean and the standard deviation of the regrouped segments are calculated as follows to provide a more accurate and continuous representation of the insect’s activity:

3 $$\begin{aligned} \mu _{f_0}^{\text {regrouped}} = \frac{\sum _{i=1}^{n} \frac{\mu _{f_0}^i}{{\sigma _{f_0}^i}^2}}{\sum _{i=1}^{n} \frac{1}{{\sigma _{f_0}^i}^2}}, \ \ \sigma _{f_0}^{\text {regrouped}} = \sqrt{\sum _{i=1}^{n} \frac{1}{{\sigma _{f_0}^i}^2}} \end{aligned}$$

### Biomass calculation

We utilized the estimated WBF to identify insect species and estimate their biomass using a reference table derived from literature mapping WBF to body mass (see Supplementary Table S2 and the references^35–108^ therein). A Pearson’s correlation test was carried out to explore the relationship between WBF and body mass (see Supplementary Figure S3), but the strength of the correlation was limited ($\rho = -0.32, P < 0.0001$). Therefore, a lookup-based algorithm was developed instead. The reference table also includes location-specific information (i.e. Europe/not Europe) and the probability of observing each insect taxon in this region, which has a placeholder value of 1.

The WBF-to-body mass mapped entries in the reference table are split into three regions as shown in Supplementary Figure S3. The dense region (WBF $\le 240$ Hz) contains 360 distinct WBF-mapped entries ($n = 360$) in the reference table. In this range, multiple candidates often share similar WBFs, leading to ambiguity in species identification. The less dense region (240–340 Hz) comprises only 14 entries ($n = 14$). Here, for a given WBF, there may be no match, a single match, or a few candidates (typically $\le 3$). The sparse region includes WBFs greater than 340 Hz, with 33 entries ($n = 33$), usually mosquitoes (Culicidae, Diptera), where a given WBF typically corresponds to a unique or nearly unique candidate. The boundaries defining the dense ($\le 240$ Hz), less dense (240–340 Hz), and sparse ($> 340$ Hz) regions were established empirically from the WBF distribution in the reference table. These thresholds reflect the increasing uniqueness of WBFs and guide the model’s handling of ambiguity at different frequency levels.

As outlined in Supplementary Algorithm S4, the body mass estimation process involves several steps. Based on the estimated WBF, candidates are found by first checking for exact matches. If no candidate is found, a range of ± 0.5 Hz around the given WBF mean is checked. If no candidate is found, the upper and lower bounds increase by 0.5 Hz until one or more candidates are found or an upper limit defined as $\frac{\max (10,\, \sigma _{f_0})}{2}$ is reached. This may then result in either 0, 1 or $>1$ candidates. If no candidate is found, and the WBF is in the dense region, the body mass is set to the median of all insect body masses in this region. This is also the case if the WBF is within the less dense region. If the mean is in the sparse region, the body mass is set to a body mass corresponding to 480 Hz in the reference table, i.e. the body mass of a mosquito. If one candidate is found, the body mass is set exactly to that of the candidate. If $>1$ candidates are found, which is often the case in the dense region, they are further filtered based on their presence/absence in Europe. This then once again results in 0, 1 or $>1$ candidates. If 0 candidates are left, the final biomass is set to 0. If 1 is left, the body mass is set to that of the found candidate. If however $>1$ candidates are left, a weighted average is calculated based on the observation probability of the given candidates. In our case this probability has been set to 1 for all European candidates.

## Field validation

This section outlines the experimental field setup and presents a comparative analysis of activity and biomass data against a conventional insect monitoring method.

### Experimental setup and data collection

**Fig. 5:**
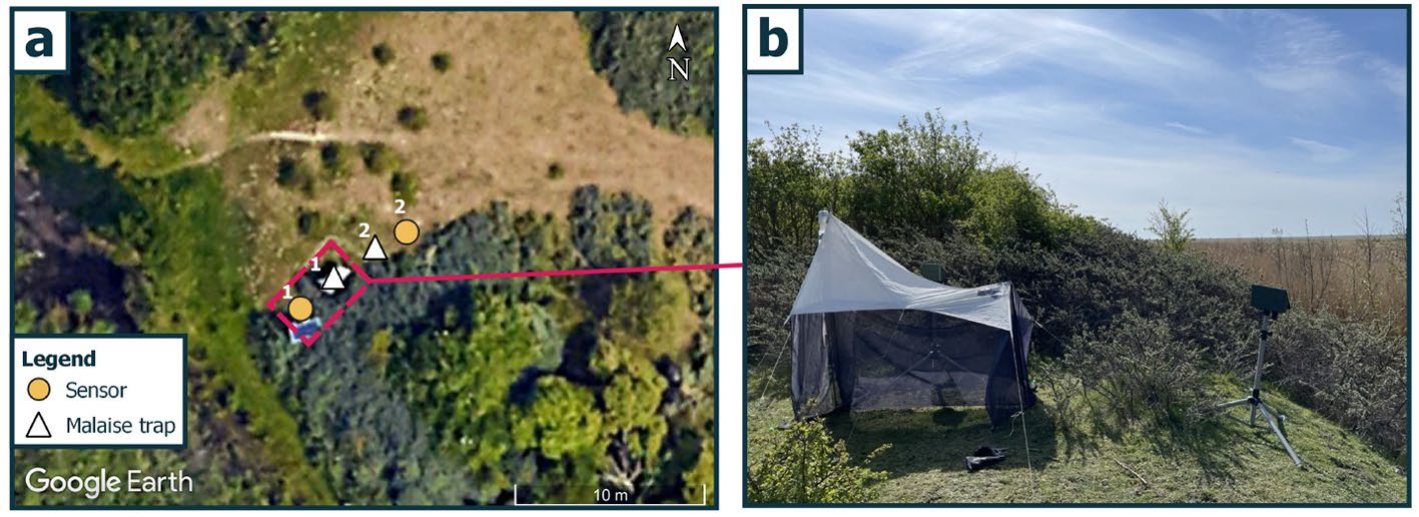
Overview of the experimental setup for field validation. (**a**) Field setup of sensors and Malaise traps. Numbers 1 and 2 denote the two sites. (**b**) Photo of site 1 with a Malaise trap (left) and a sensor (right). Basemap by Google Earth. The figure was made using QGIS^109^.

The sensor was field-tested alongside a conventional monitoring method for flying insects, the Townes Malaise trap^26^, at a nature reserve in the Capital Region of Denmark (12.5305 E, 55.5933 N). The experimental setup included two sensors and two Malaise traps, deployed in close proximity to each other, referred to as site 1 and site 2 (sampling period: June 17, 2024, to September 12, 2024) (Fig. 5).

#### Samples

Since the sensors are solar powered, they monitor insect counts and biomass continuously from one hour before sunrise until one hour after sunset, which had a minor variation throughout the sampling season. These counts and biomass values were then summed up corresponding to each Malaise trapping period.

The Malaise traps were emptied daily from 17/06/2024 to 21/06/2024 and from 24/06/2024 to 28/06/2024, whilst from 11/07/2024 to 12/09/2024, the traps were emptied twice a week on Mondays and Thursdays. 70 percent isopropyl alcohol was used as a preservative both in the container in the trap and to store the samples afterwards. Within the first 24 hours, the collected material was drained from the isopropyl alcohol using a fine-mesh sieve and spread out on absorbent paper for five minutes to allow the alcohol to evaporate^110^. All winged insect specimens were thereafter counted. Wingless insects such as ants (Formicidae, Hymenoptera) and nymphal stages of hemimetabolous insects were excluded from the samples to give a more precise comparison of the counts. A total of 55,443 specimens were collected for the two Malaise traps in the sampling period, which aligns with findings from similar environments^111^. See Supplementary Table S6 for an overview of insect counts and biomass for sensors and Malaise traps.

### Data analysis

Since only one variable was normally distributed (see Supplementary Table S9), Spearman’s correlation tests were conducted on both biomass and insect count data between Malaise traps and sensors. Malaise-Malaise and sensor-sensor correlation tests were also conducted for both biomass and insect count data. To compare correlation coefficients between these two latter correlations, Fischer’s Z-transformation tests^112^ were conducted. All statistical tests were two-tailed, and statistical significance was set at $\alpha = 0.05$. All statistical analyses have been conducted using RStudio^113,114^.

## Results

**Fig. 6:**
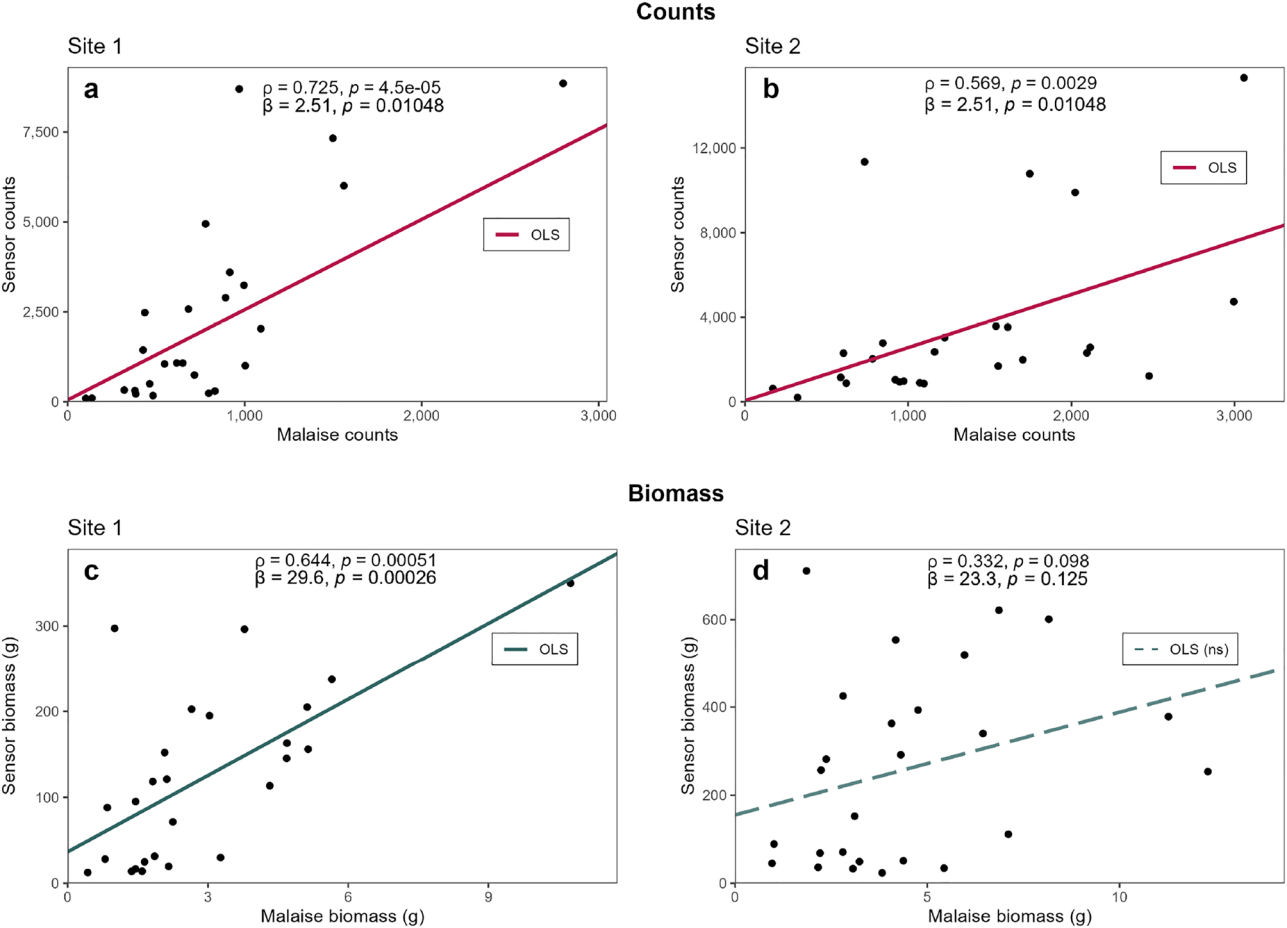
Sensor counts and biomass compared to Malaise trap counts and biomass for both sites 1 and 2. OLS lines are plotted (dashed = non-significant), along with Spearman’s correlation coefficients $\rho$ and OLS slopes $\beta$ and their corresponding *P*-values. $n = 26$. (**a**) Site 1 counts. (**b**) Site 2 counts. (**c**) Site 1 biomass. (**d**) Site 2 biomass.

**Fig. 7:**
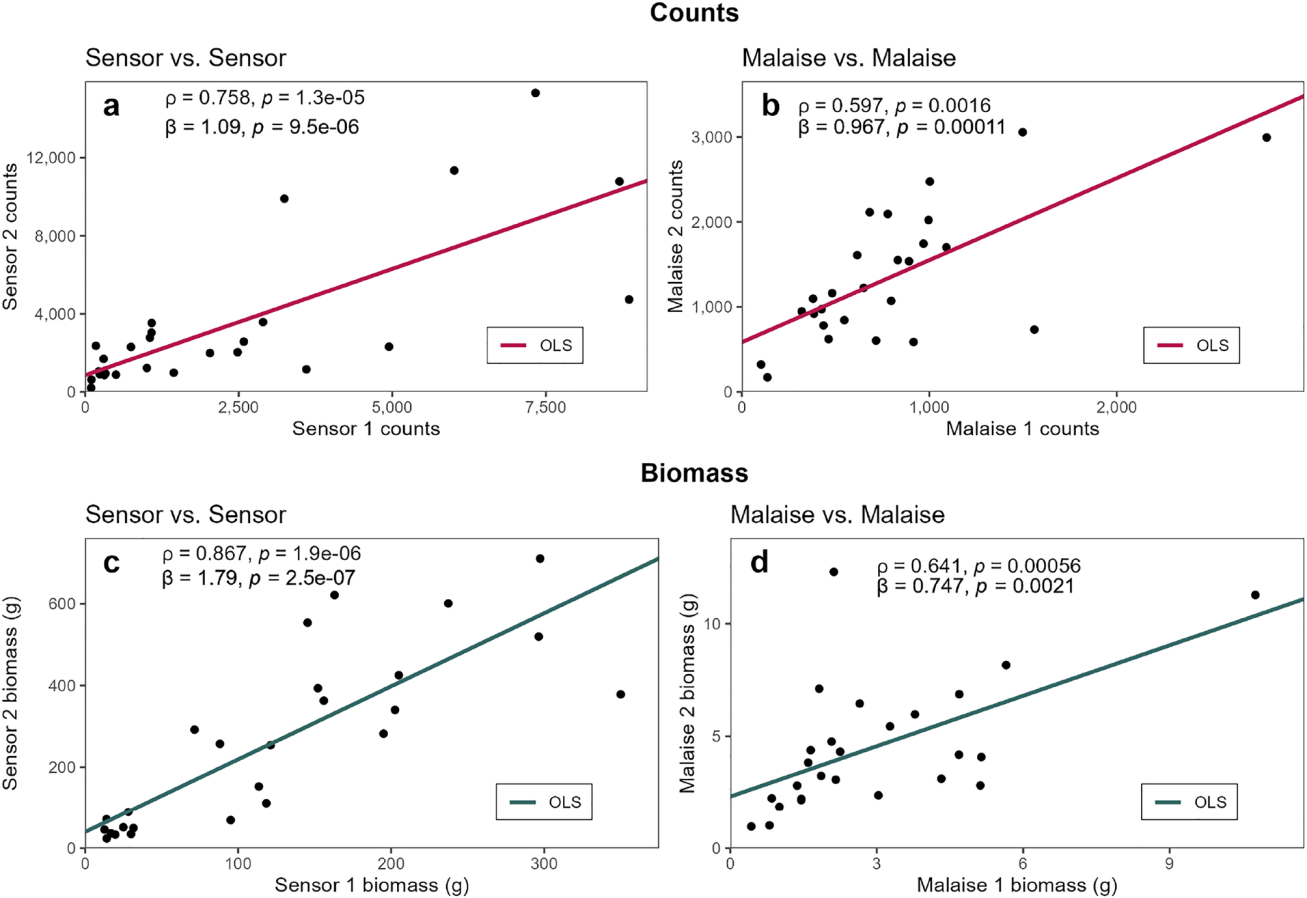
Sensor-Sensor and Malaise-Malaise comparison of counts and biomass for both sites 1-2. OLS lines are plotted, along with Spearman’s correlation coefficients $\rho$ and OLS slopes $\beta$ and their corresponding *P*-values. $n = 26$. (**a**) Comparison of counts between sensors 1 and 2. (**b**) Comparison of insect counts between Malaise traps 1 and 2. (**a-b**) Fischer’s Z-score = 1.029 ($P = 0.304$). (**c**) Comparison of insect biomass (g) between sensors 1 and 2. (**d**) Comparison of insect biomass (g) between Malaise traps 1 and 2. (**c-d**) Fischer’s Z-score = 1.907 ($P = 0.057$).

The correlation test results comparing sensor and Malaise counts are shown in Fig. 6a-b, as well as for biomass in Fig. 6c-d. The correlation test results for sensor-sensor counts and Malaise-Malaise counts are shown in Fig. 7a-b, as well as for biomass in Fig. 7c-d. Fischer’s Z-transformation test results are shown in Fig. 7. Ordinary least squares (OLS) linear models have been plotted for all figures to illustrate the tendency between variables.

## Discussion

The field validation results demonstrate the effectiveness of our signal processing approach and probe design in real-world conditions. The differential electric field measurement technique, with its distinctive dual-probe configuration creating a symmetry plane (as shown in Fig. 3), successfully mitigated environmental noise while maintaining sensitivity to insect activity. This approach efficiently suppressed far-field signals such as radar, communication, and meteorological events. However, power line noise from nearby sources presented a challenge to common mode rejection due to their physical length and varying distances, requiring specialized filtering techniques. Despite these challenges, the CNN-based detection algorithm demonstrated robust performance in distinguishing insect signals from background interference, considering the limited labeled dataset and its geographical bias (sensors used for training and evaluating the model were mainly installed in Northern Europe), which may limit model performance in other ecological contexts. This necessitates expansion of training datasets across other geographical regions. The post-processing signal aggregation method improved continuity in tracking individual insect events, enhancing the accuracy of both count and biomass estimates. Notably, the probabilistic YIN algorithm’s extraction of WBFs proved critical for taxonomic identification. An alternative and potentially more robust approach could involve joint modeling of insect detection and WBF estimation using object detection frameworks applied to spectrograms–mimicking the way human annotators identify insect events visually in spectrograms. By detecting and localizing time-frequency patterns simultaneously, such models could capture subtle spectral features and improve resilience to noise and overlapping events.

As shown in Fig. 6a-b, the counts between sensors and Malaise traps were significantly correlated for both sites, with a stronger correlation at site 1 ($\rho = 0.725$, $P < 0.0001$), compared to site 2 ($\rho = 0.569$, $P = 0.0029$). A similar trend was observed for the biomass estimates (Figs. 6c-d), though the correlation was weaker for site 1 ($\rho = 0.644$, $P = 0.00051$) and not significant for site 2 ($\rho = 0.332$, $P = 0.098$). Additionally, the OLS slopes in Fig. 6a-b indicate that the sensors recorded approximately three times higher insect counts compared to the Malaise traps. This difference was even more pronounced for biomass, where the sensor estimates were roughly 26 times higher (Fig. 6c-d). These discrepancies can largely be attributed to fundamental differences between the two monitoring methods. The sensor employs a passive, non-invasive detection mechanism, allowing individual insects to pass through the detection area multiple times, potentially inflating the observed abundance and biomass. In contrast, the Malaise trap captures insects only once and permanently removes them from the population. Additionally, since sensor measurements were limited to daytime, the observed differences might be even greater if the sensors had been active at night as well. In fact, a study by Wong and Didham^115^ suggests that, although highly variable, average global insect activity is higher during nighttime, albeit more pronounced in the tropics and in aquatic taxa.

While the difference in overall measurements can be explained by the differing methodologies, it does not wholly account for the much larger discrepancy in biomass values. This can be attributed to the variability in the WBF-to-biomass mapping, particularly in the lower frequency ranges where multiple candidate species may exist (Supplementary Figure S3). This likely contributes to the observed measurement differences between sensor and Malaise trap data, beyond the methodological distinctions discussed above. Future research should therefore explore other parameters, in addition to WBF alone, that could be important in distinguishing between insect taxa. Expanding on the geographic location probabilities for the reference table would be beneficial as well. Furthermore, Malaise traps are known to underestimate certain taxa^19^, particularly Coleoptera, as beetles frequently collide with the trap mesh and escape before capture^116^. Given that beetles are heavily schlerotized and therefore proportionally heavier than other insects of the same size^117^, this bias likely leads to an underestimation of biomass in the Malaise trap data. Additionally, uncertainties in the insect weight data from the reference table (Supplementary Table S2 and the references^35–108^ therein) may contribute to discrepancies in the biomass estimation algorithm, as literature values often lack clarity on whether the weights were recorded in dry, wet, or fresh states. While all the above-mentioned uncertainties explain much of the variation, such a large discrepancy in biomass highlights the need for further calibration before this system can be used for absolute biomass estimation. One approach that could prove useful is exploring the weak, yet significant negative correlation between WBF and body mass (Supplementary Figure S3). This could be used to develop a predictive model, potentially in combination with our lookup-based method.

Another point of discussion for the biomass estimate is the minimum WBF threshold of 20 Hz. Since WBF and body mass is negatively correlated, this results in heavy insect candidates being filtered out before biomass estimation. However, candidates below 20 Hz in the reference table (Supplementary Table S2) almost only constitute damsel- and dragonflies (Odonata) as well as bombycoid moths and true butterflies (Bombycoidea and Papillionoidea, Lepidoptera) – groups that Malaise traps very rarely collect^19^. Therefore this cut-off at 20 Hz should, hypothetically, not be an issue when comparing biomass between the two methods.

When comparing measurements between two sensors and between two Malaise traps deployed under similar conditions (Fig. 7), the sensor-sensor correlations (Fig. 7a and c) were higher than the Malaise-Malaise correlations (Fig. 7b and d). This suggests that the sensor system provides more consistent measurements. However, the difference was not statistically significant for either counts (Z-score = 1.029, $P = 0.304$) or biomass (Z-score = 1.907, $P = 0.057$). Although the *P*-value for biomass does not meet the conventional significance threshold ($\alpha = 0.05$), its proximity to significance ($P = 0.057$) suggests a potential difference that warrants further investigation.

Another consideration when designing insect monitoring devices is coloration. A study by Campbell and Hanula^118^ demonstrates that adding color panels to Malaise traps has an effect on pollinator captures. Other studies using different pan trap colors reveal that yellow^119,120^ and white^121^ pan traps seem to be the most efficient. Therefore it is reasonable to presume that the dark green color coating of the sensor (Fig. 5b) deployed in this study is less efficient at attracting insects compared to yellow and white colored objects, and therefore arguably less biased in its detectability of flying insects. The Malaise trap, conversely, has white roofing (Fig. 5b), which could have a positive bias in attracting insects who prefer white. In fact, a study on Malaise trap types and butterflies (Lepidoptera) by Hoffmann et al.^122^ reveals that white-roofed Malaise traps catch higher biomass and richness of butterflies compared to black-roofed ones.

## Conclusion

Effective insect monitoring is essential for the conservation of nature, as well as for assessing ecosystem health. Traditional methods like Malaise trapping are labor-intensive and offer limited temporal resolution. In contrast, the sensor-based approach in this study enables continuous data collection while reducing manual effort. The moderate to strong correlations between sensor and Malaise trap data suggests that the sensor can estimate insect activity and biomass with moderate reliability, though further validation is needed for future consistency and scalability in long-term monitoring efforts. Beyond efficiency, these sensors mitigate the ethical concerns of lethal sampling. In this study alone, 55,443 insects (Supplementary Table S6) were killed in two Malaise traps. Reducing reliance on such methods would minimize disruption to local insect populations while providing high-quality data for ecological research. Further validation across diverse habitats will be crucial for optimizing accuracy and broadening the applicability of this technology. While the biomass algorithm needs refinement and further validation, these findings offer a promising passive method for monitoring insects while providing valuable insights into insect behavior and populations without the need for active signal generation or physical, invasive trapping methods.

## Supplementary Information

Supplementary Information.

## Data Availability

All data generated or analyzed during this study are included in this published article (and its Supplementary Materials).
