# Supplementary material for: Automated insect detection and biomass monitoring via AI and electrical field sensor technology
Source: Sci Rep. 2025 Aug 14;15:29858. doi: 10.1038/s41598-025-15613-5 (PMC12354830; doi:10.1038/s41598-025-15613-5)
Supplement: Supplementary file 1 — Supplementary Information. [file 41598_2025_15613_MOESM1_ESM.pdf]

Supplementary Materials
for
Automated Insect Detection and Biomass Monitoring via AI and Electrical Field Sensor Technology

Freja Balmer Odgaard[1,*], Páll Vang Kjærbo[1], Amir Hossein Poorjam[1], Khaled Hechmi[1], Rubens Monteiro Luciano[1], and Niels Krebs[1]

[1]FaunaPhotonics, Oceanvej 1, Copenhagen, 2150, Denmark
[*]frod@faunaphotonics.com

S1: Data processing algorithm

Input: 1-minute audio signals from cloud storage
Output: Estimated insect biomass
Step 1: Load Audio
Load raw audio data
Step 2: Power line Noise Cancellation
if power line noise is detected **then**
    Apply filtering to remove it
end if
Step 3: Signal Segmentation
Divide the signal into $N = 60$ segments
for $i = 1$ to N **do**
    Step 4.1: CNN-based Insect Presence Prediction
    Pass segment i to the CNN to compute a prediction score
    if insect presence is indicated **then**
        Step 4.2: $\mathbf{F_0}$ Estimation
        Estimate F_0 and compute its mean ($\mu^i_{f_0}$) and standard deviation ($\sigma^i_{f_0}$)
        if $\mu^i_{f_0}$ and $\sigma^i_{f_0}$ meet threshold criteria **then**
            Mark segment i as a valid insect event
        else
            Discard segment i
        end if
    else
        Discard segment i
    end if
end for
Step 5: Event Aggregation
Group adjacent valid events
Step 6: Data Collection
Aggregate data from valid events
Step 7: Biomass Estimation
Apply biomass model to compute the final estimate
    return Estimated insect biomass

S2: Reference table for WBF and body mass

Reference table containing insect body mass (g), state of specimens when weighed (dry/wet/fresh), and their wing beat frequency (WBF) for various species, genera, families, and orders of insects. The † sign denotes internal FaunaPhotonics measurements. The table is ordered by increasing WBF.

Order	Family	Species	Body Mass (g)	Wet/Dry/Fresh weight	WBF (Hz)	Europe (Yes/No)[35]
Lepidoptera	Pieridae	*Pieris napi*	0.037[36]		6[36]	Yes
Lepidoptera					6[37]	Yes
Lepidoptera	Saturniidae	*Saturnia pyri*	1.89[36]		8[36]	Yes
Lepidoptera	Saturniidae	*Philosamia cynthia*	0.605[36]		8[36]	Yes
Lepidoptera	Papilionidae	*Papilio podalirius*	0.3[36]		10[36]	Yes
Lepidoptera	Nymphalidae	*Argynnis pandora*	0.278[36]		10[36]	Yes
Lepidoptera	Pieridae	*Pieris brassicae*	0.144[36]		10[36]	Yes
Lepidoptera	Nymphalidae	*Vanessa atalanta*	0.134[36]		10[36]	Yes
Lepidoptera	Pieridae	*Pieris brassicae*	0.127[36]		12[36]	Yes
Lepidoptera	Pieridae	*Pieris brassicae*	0.0691[38]		12[38]	Yes
Lepidoptera	Saturniidae	*Automeris belti*	0.665[36]		14[36]	No
Lepidoptera	Nymphalidae	*Aphantopus hyperantus*	0.0374[38]		16[38]	Yes
Lepidoptera	Saturniidae	*Automeris jaciinda*	0.298[36]		17[36]	No
Lepidoptera	Saturniidae	*Eacles imperialis*	1.105[36]		17[36]	No
Lepidoptera	Saturniidae	*Automeris fieldi*	0.394[36]		17[36]	No
Odonata	Calopterygidae	*Calopteryx virgo*	0.1457[38]		17[38]	Yes
Lepidoptera	Nymphalidae	*Vanessa io*	0.195[36]		18[36]	Yes
Lepidoptera	Bombycidae	*Bombyx mori*	0.595[36]		18[36]	No
Odonata					18[37]	Yes
Lepidoptera	Saturniidae	*Automeris zugana*	0.428[36]		19[36]	No
Neuroptera			0.009[39]	Wet	19[37]	Yes
Odonata	Calopterygidae	*Calopteryx splendens*	0.1092[38]		19[38]	Yes
Lepidoptera	Nymphalidae	*Vanessa cardui*	0.173[36]		20[36]	Yes
Lepidoptera	Sphingidae	*Chelonia villica*	0.165[36]		20[36]	Yes
Odonata	Aeshnidae	*Anax junius*	0.82[36]		20[36]	No
Odonata	Libellulidae	*Libellula depressa*	0.245[36]		20[36]	Yes
Odonata	Libellulidae	*Orthetrum coerulescens*	0.248[36]		20[36]	Yes
Odonata	Aeshnidae	*Aeshna sp.*	0.611[36]		20[36]	Yes
Lepidoptera	Lepedoptera	*Rhodocera rhamni*	0.107[36]		21[36]	Yes
Odonata	Libellulidae	*Libellula quadrimaculata*	0.307[36]		21[36]	Yes
Odonata	Libellulidae	*Sympetrum meridionale*	0.281[36]		21[36]	Yes
Odonata	Aeshnidae	*Nasiaeschna pentacantha*	0.43[36]		21[36]	No

Table continued from previous page

Order	Family	Species	Body Mass (g)	Wet/Dry/Fresh weight	WBF (Hz)	Europe (Yes/No)[35]
Lepidoptera	Saturniidae	*Hyperchiria nausica*	0.2[36]		21[36]	No
Odonata	Libellulidae	*Pantala flavescens*	0.308[36]		22[36]	Yes
Lepidoptera	Notodontidae	*Notodonta dictaea*	0.201[36]		22[36]	Yes
Lepidoptera	Satyridae	*Coenonympha pamphilus*	0.046[36]		22[36]	Yes
Lepidoptera	Saturniidae	*Syssphinx molina*	1.63[36]		22[36]	No
Lepidoptera	Sphingidae	*Manduca corallina*	1.6[36]		22[36]	No
Odonata	Aeshnidae	*Anax formosus*	1.2[36]		22[36]	No
Lepidoptera	Geometridae	*Geometra papilionaria*	0.1071[38]		22[38]	Yes
Lepidoptera	Saturniidae	*Automerina auletes*	0.72[36]		23[36]	No
Odonata	Libellulidae	*Tramea Carolina*	0.358[36]		23[36]	No
Lepidoptera	Sphingidae	*Erinnyis ello*	1.21[36]		23[36]	No
Lepidoptera	Saturniidae	*Automeris hamata*	0.564[36]		23[36]	No
Lepidoptera	Saturniidae	*Adeloneivaia boisduvalii*	0.839[36]		24[36]	No
Odonata	Libellulidae	*Pachydiplax longipenms*	0.178[36]		24[36]	No
Lepidoptera	Noctuidae	*Triphaena pronuba*	0.485[36]		24[36]	Yes
Neuroptera			0.009[39]	Wet	24[37]	Yes
Lepidoptera	Noctuidae	*Venilia macularia*	0.021[36]		25[36]	Yes
Odonata	Macromiidae	*Macromia taeniolata*	0.93[36]		25[36]	No
Odonata	Corduliidae	*Somatochlora tenebrosa*	0.352[36]		25[36]	No
Neuroptera	Chrysopidae	*Chrysoperla carnea*	0.0065[38]		25[38]	Yes
Blattodea	Blattidae	*Periplanela americana*	1.555[36]		26[36]	Yes
Odonata	Libellulidae	*Tramea lacerata*	0.382[36]		26[36]	No
Odonata	Libellulidae	*Libellula pulchella*	0.508[36]		26[36]	No
Odonata	Corduliidae	*Tetragoneuria cynosura*	0.165[36]		27[36]	No
Lepidoptera	Lymantriidae	*Lymantria clispar*	0.101[36]		27[36]	Yes
Lepidoptera	Nymphalidae	*Polygonia c-album*	0.1145[38]		27[38]	Yes
Trichoptera	Phryganeidae	*Phryganea grandis*	0.159[38]		27[38]	Yes
Trichoptera			0.159[38]		27[38]	Yes
Lepidoptera	Lymantriidae	*Calliteara pudibunda*	0.237[36]		28[36]	Yes
Lepidoptera	Sphingidae	*Manduca corallina*	1.618[36]		28[36]	No
Odonata	Libellulidae	*Erythemis simplicicollis*	0.176[36]		28[36]	No
Mecoptera	Panorpidae	*Panorpa communis*	0.03[36]		28[36]	Yes
Odonata					28[37]	Yes
Lepidoptera	Sphingidae	*Manduca nistica*	2.704[36]		29[36]	No
Lepidoptera	Sphingidae	*Acherontia atropos*	2.2403[38]		29[38]	Yes
Lepidoptera	Geometridae	*Xanthorhoe montanata*	0.0133[38]		29[38]	Yes
Lepidoptera	Sphingidae	*Laothoe populi*	0.8449[38]		29[38]	Yes

Table continued from previous page

Order	Family	Species	Body Mass (g)	Wet/Dry/Fresh weight	WBF (Hz)	Europe (Yes/No)[35]
Odonata	Macromiidae	*Macromia georgina*	0.545[36]		30[36]	No
Neuroptera			0.009[39]	Wet	30[37]	Yes
Lepidoptera	Noctuidae	*Hypena proboscidalis*	0.0565[38]		30[38]	Yes
Odonata	Libellulidae	*Plathemis lydia*	0.365[36]		31[36]	No
Odonata	Aeshnidae	*Aeshna grandis*	1.2296[38]		31[38]	Yes
Lepidoptera	Saturniidae	*Hylesia spp.*	0.168[36]		32[36]	
Lepidoptera	Geometridae	*Idaea aversata*	0.0303[38]		32[38]	Yes
Odonata			0.27[38]		32[38]	Yes
Lepidoptera	Pterophoridae	*Pterophorus pentadactyla*	0.0114[38]		32[38]	Yes
Odonata	Aeshnidae	*Brachytron pratense*	0.557[36]		33[36]	Yes
Coleoptera	Lucanidae	*Ljicanus cervus*	2.6[36]		33[36]	No
Lepidoptera	Sphingidae	*Manduca lefeburei*	0.571[36]		33[36]	No
Lepidoptera	Erebidae	*Eilema lurideola*	0.0258[38]		33[38]	Yes
Lepidoptera	Plutellidae	*Plutella xylostella*	0.0019[40]	Fresh	35†	Yes
Odonata	Coenagrionidae	*Coenagrion puella*	0.0316[38]		36[38]	Yes
Odonata	Coenagrionidae	*Coenagrion puella*	0.0374[38]		36[38]	Yes
Odonata	Coenagrionidae	*Coenagrion puella*	0.0277[38]		37[38]	Yes
Odonata	Aeshnidae	*Aeshna mixta*	0.53[36]		38[36]	Yes
Odonata					38[37]	Yes
Odonata	Libellulidae	*Orthetrum cancellatum*	0.4176[38]		38[38]	Yes
Lepidoptera	Sphingidae	*Oryba achemenides*	2.809[36]		39[36]	No
Lepidoptera			0.203[38]		39[38]	Yes
Lepidoptera	Crambidae	*Chrysoteuchia culmella*	0.109[38]		40[38]	Yes
Odonata	Libellulidae	*Sympetrum striolatum*	0.1595[38]		40[38]	Yes
Lepidoptera	Crambidae	*Anania hortulata*	0.0293[38]		40[38]	Yes
Lepidoptera	Noctuidae	*Agrostis exclaniationis*	0.133[36]		41[36]	Yes
Lepidoptera	Sphingidae	*Madoryx oeclus*	1.699[36]		41[36]	No
Lepidoptera	Saturniidae	*Adeloneivaia subangulata*	0.487[36]		41[36]	No
Lepidoptera	Geometridae	*Pasiphila rectangulata*	0.0107[38]		41[38]	Yes
Odonata	Gomphidae	*Ophiogomphus serpentiniis*	0.312[36]		42[36]	Yes
Hemiptera	Cicadidae	*Cicada sp.*	0.752[36]		42[36]	Yes
Lepidoptera	Tortricidae	*Adoxophyes orana*	0.01532[41]	Fresh	43†	Yes
Lepidoptera					43[37]	Yes
Lepidoptera	Sphingidae	*Xylophones pluto*	0.829[36]		45[36]	No
Neuroptera	Hemerobiidae	*Wesmaelius subnebulosis*	0.0026[38]		45[38]	Yes
Coleoptera	Melolonthidae	*Melolontha vulgaris*	0.961[36]		46[36]	Yes
Neuroptera	Hemerobiidae	*Hemerobius humulinus*	0.0021[38]		46[38]	Yes

Table continued from previous page

Order	Family	Species	Body Mass (g)	Wet/Dry/Fresh weight	WBF (Hz)	Europe (Yes/No)[35]
Lepidoptera	Noctuidae	*Orthosia gothica*	0.1253[38]		47[38]	Yes
Diptera	Tipulidae	*Tipula gigantea*	0.069[36]		48[36]	Yes
Lepidoptera	Noctuidae	*Plusia gamma*	0.144[36]		48[36]	Yes
Lepidoptera	Zygaenidae	*Zygaena filipendulae*	0.127[36]		48[36]	Yes
Lepidoptera	Sphingidae	*Xylophanes libya*	0.559[36]		48[36]	No
Lepidoptera	Sphingidae	*Pachygonidia drucei*	0.702[36]		48[36]	No
Diptera	Tipulidae				48[42]	Yes
Mecoptera	Panorpidae	*Panorpa communis*	0.0398[38]		48[38]	Yes
Mecoptera			0.0398[38]		48[38]	Yes
Lepidoptera	Yponomeutidae	*Yponomeuta spp.*			50†	Yes
Neuroptera	Hemerobiidae	*Micromus angulatus*	0.0059[38]		50[38]	Yes
Lepidoptera	Tortricidae	*Plodia interpunctella*	0.004305[43]	Fresh	50†	Yes
Lepidoptera	Noctuidae	*Helicoverpa armigera*	0.13505[44]	Fresh	52†	Yes
Diptera	Tipulidae	*Tipula sp.*	0.03[36]		52[36]	Yes
Lepidoptera	Tortricidae		0.0055[38]		52[38]	Yes
Neuroptera			0.0035[38]		52[38]	Yes
Lepidoptera	Sphingidae	*Deilephila elpenor*	0.5281[38]		53[38]	Yes
Lepidoptera	Tortricidae	*Pandemis cerasana*	0.0124[38]		54[38]	Yes
Neuroptera	Hemerobiidae	*Wesmaelius sp.*	0.0033[38]		54[38]	Yes
Lepidoptera	Lasiocampidae	*Poecilocampa populi*	0.112[36]		55[36]	Yes
Lepidoptera	Sphingidae	*Enyo ocypete*	0.388[36]		56[36]	No
Diptera	Tipulidae				57[42]	Yes
Lepidoptera	Crambidae		0.0059[38]		57[38]	Yes
Lepidoptera	Lasiocampidae	*Malacosoma americanum*	0.088[36]		58[36]	No
Diptera	Tipulidae	*Tipula oleracea*			59†	Yes
Diptera	Tipulidae	*Tipula sp.*	0.0676[38]		59[38]	Yes
Lepidoptera	Zygaenidae	*Zygaena sp.*	0.0804[38]		60[38]	
Lepidoptera	Sphingidae	*Perigonia lusca*	0.638[36]		62[36]	No
Hymenoptera	Ichneumonidae	*Ophion luteus*	0.033[36]		62[36]	Yes
Coleoptera	Melolonthidae	*Melolontha vulgaris*	0.597[36]		62[36]	Yes
Lepidoptera	Tortricidae	*Cydia pomonella*	0.0265[45]	Fresh	62†	Yes
Lepidoptera	Tortricidae	*Pseudargyrotoza conwagana*	0.0044[38]		64[38]	Yes
Lepidoptera	Tortricidae	*Lobesia abscisana*	0.0076[38]		64[38]	Yes
Odonata	Coenagrionidae	*Ischnura elegans*	0.033[46]	Fresh	65†	Yes
Lepidoptera	Tortricidae	*Lobesia botrana*	0.00655[47]	Fresh	66†	Yes
Hemiptera	Tessaratomidae	*Tessaratoma javanica*	0.926[36]		66[36]	
Diptera	Tipulidae	*Nephrotoma quadrifaria*	0.0181[38]		67[38]	Yes

Table continued from previous page

Order	Family	Species	Body Mass (g)	Wet/Dry/Fresh weight	WBF (Hz)	Europe (Yes/No)[35]
Coleoptera	Cantharidae	*Telephorus fuscipes*	0.109[36]		72[36]	Yes
Lepidoptera	Sphingidae	*Macroglossa stellatarum*	0.282[36]		73[36]	Yes
Diptera	Trichoceridae	*Trichocera sp.*	0.0012[36]		74[36]	Yes
Coleoptera	Chrysomelidae	*Agelastica alni*	0.03115[48]		75†	Yes
Coleoptera	Cantharidae	*Rhagonycha fulva*	0.0183[38]		75†	Yes
Ephemeroptera	Baetidae	*Centroptilum luteolum*	0.0027[38]		75[38]	Yes
Ephemeroptera			0.0027[38]		75[38]	Yes
Coleoptera	Scarabaeidae	*Amphimallon solstitiale*	0.291[36]		78[36]	Yes
Coleoptera	Coccinellidae	*Harmonia axyridis*	0.0283[38]		79[38]	Yes
Diptera	Tipulidae	*Nephrotoma flavescens*	0.0118[38]		79[38]	Yes
Coleoptera	Cantharidae	*Rhagonycha fulva*	0.0183[38]		79[38]	Yes
Hymenoptera	Ichneumonidae	*Amblyteles armatorius*	0.0569[49]		80[49]	No
Coleoptera	Cerambycidae		0.142[36]		80[36]	Yes
Lepidoptera	Sphingidae	*Macroglossa bombyliformis*	0.189[36]		80[36]	Yes
Hemiptera	Aphididae	*Acyrthosiphon kondoi*	0.000702[36]		81[36]	Yes
Lepidoptera					81[37]	Yes
Lepidoptera	Sphingidae	*Macroglossa stellatarum*	0.345[36]		85[36]	No
Coleoptera	Chrysomelidae	*Psylliodes chrysocephala*			85†	Yes
Hymenoptera	Apidae	*Exaerete frontalis*	0.699[50]		86[50]	No
Coleoptera	Cetoniidae	*Cetonia aurata*	0.537[36]		86[36]	Yes
Coleoptera	Cerambycidae	*Rutpela maculata*	0.1026[38]		86[38]	Yes
Hymenoptera	Apidae	*Exaerete frontalis*	0.644[36]		87[36]	No
Hymenoptera	Tenthredinidae	*Athalia scutelariae*	0.0132[38]		87[38]	Yes
Hemiptera	Aphrophoridae	*Neophilaenus sp.*			89†	Yes
Hymenoptera	Apidae	*Bombus lapidarius*	0.495[36]		90[36]	Yes
Hymenoptera	Tenthredinidae	*Athalia rosae*	0.0104[51]	Fresh	90†	Yes
Diptera	Phoridae		0.0005†	Wet	90[42]	Yes
Hemiptera	Aphididae	*Mysus persicae*	0.000334[36]		90[36]	Yes
Hemiptera	Aleyrodidae	*Aleyrodes proletella*	6.3e-05[52]		92†	No
Hemiptera	Pentatomidae	*Halyomorpha halys*	0.09843[53]	Fresh	92†	Yes
Diptera	Heleomyzidae				92[42]	Yes
Coleoptera	Scarabaeidae	*Aphodius sp.*	0.0327[38]		93[38]	Yes
Coleoptera	Cerambycidae	*Leptura quadrifasciata*	0.1173[38]		93[38]	Yes
Neuroptera	Hemerobiidae	*Micromus sp.*	0.0003[38]		94[38]	Yes
Diptera	Tipulidae		0.002[38]		94[38]	Yes
Coleoptera	Curculionidae	*Hypera meles*			95†	Yes
Diptera	Tabanidae	*Tabanus bovinus*	0.276[36]		96[36]	Yes
Hemiptera	Pentatomidae	*Pentatoma rufipes*	0.1397[38]		96[38]	Yes

Table continued from previous page

Order	Family	Species	Body Mass (g)	Wet/Dry/Fresh weight	WBF (Hz)	Europe (Yes/No)[35]
Coleoptera			0.0539[38]		97[38]	Yes
Hymenoptera	Apidae	*Eulaema meriana*	0.94[36]		98[36]	No
Hymenoptera	Cimbicidae	*Cimbex sp.*			99†	Yes
Hemiptera	Aphididae	*Uroleucon cirsii*	0.0015[38]		99[38]	Yes
Hemiptera	Scutelleridae	*Chrysocoris purpureus*	0.264[36]		100[36]	No
Diptera	Tabanidae	*Dasyramphis atra*	0.233[36]		100[36]	Yes
Hemiptera	Aphididae	*Brevicoryne brassicae*	0.00084[54]	Fresh	100†	Yes
Hymenoptera	Vespidae	*Vespa crabro*	0.567[36]		100[36]	Yes
Coleoptera	Scarabaeidae	*Aphodius sp.*	0.1095[38]		101[38]	Yes
Diptera	Lonchopteridae				102[42]	Yes
Coleoptera	Coccinellidae	*Propylea 14-punctata*	0.0105[38]		102[38]	Yes
Diptera	Tabanidae				102[42]	Yes
Coleoptera	Scarabaeidae	*Aphodius sp.*	0.0929[38]		103[38]	Yes
Coleoptera	Rutelidae	*Popillia japonica*	0.0501[55]		104[37]	No
Hymenoptera	Vespidae	*Vespa crabro*	0.597[36]		104[36]	Yes
Hemiptera	Aphididae	*Aphis fabae*	0.000641[56]		104†	Yes
Diptera	Scathophagidae	*Scathophaga stercoraria*	0.0224[38]		104[38]	Yes
Hemiptera	Aphididae	*Myzus persicae*	0.0004[57]	Fresh	104†	Yes
Hemiptera	Aphididae	*Aphis fabae*	0.000411[36]		104[36]	Yes
Hymenoptera	Vespidae	*Polistes dominula*	0.0898[58]	Fresh	105†	Yes
Hymenoptera	Apidae	*Eulaema meriana*	0.875[50]		105[50]	No
Diptera	Therevidae				106[42]	Yes
Coleoptera	Rutelidae	*Popillia japonica*	0.1504[59]		107†	Yes
Hemiptera	Miridae		0.0119[38]		108[38]	Yes
Hymenoptera	Apidae	*Eulaema bombiformis*	0.983[50]		109[50]	No
Diptera	Stratiomyidae				109[42]	Yes
Hymenoptera	Vespidae	*Vespa germanica*	0.187[36]		110[36]	Yes
Hymenoptera	Ichneumonidae		0.0233[38]		110[38]	Yes
Hymenoptera	Andrenidae	*Andrena vaga*	0.0957[60]	Fresh	112†	Yes
Coleoptera	Oedemeridae	*Oedemera nobilis*	0.021[38]		112[38]	Yes
Hymenoptera	Vespidae	*Vespa crabro*	0.4775[61]		113†	Yes
Diptera	Syrphidae	*Syrphus grossulariae*	0.02[36]		114[36]	Yes
Diptera	Psychodidae				114[42]	Yes
Hemiptera	Miridae	*Lygus rugulipennis*	0.014[38]		115[38]	Yes
Coleoptera	Nitidulidae	*Brassicogethes aeneus*	0.0014[62]		115†	Yes
Hemiptera			0.0226[38]		116[38]	Yes
Hymenoptera	Braconidae				117†	Yes
Hymenoptera	Tenthredinidae	*Cladius pectinicornis*			117†	Yes

Table continued from previous page

Order	Family	Species	Body Mass (g)	Wet/Dry/Fresh weight	WBF (Hz)	Europe (Yes/No)[35]
Orthoptera	Acrididae	*Chorthippus brunneus*	0.16[63]	Fresh	118†	Yes
Hemiptera	Aphididae	*Aphis nerii*	0.000467[36]		118[36]	Yes
Diptera	Syrphidae	*Volucella plumata*	0.124[36]		120[36]	Yes
Diptera	Syrphidae	*Volucella pellucens*	0.073[36]		120[36]	Yes
Diptera	Syrphidae	*Chrysotoxum bicincta*	0.075[36]		120[36]	Yes
Hemiptera	Miridae	*Lygocoris pabulinus*	0.00556[64]	Fresh	120†	Yes
Hymenoptera	Sphecidae	*Ammophila sabulosa*	0.045[36]		120[36]	No
Hemiptera	Miridae		0.0048[38]		120[38]	Yes
Coleoptera	Tenthredinidae	*Ceutorhynchus obstrictus*			122†	Yes
Hemiptera	Cicadellidae	*Scaphoideus titanus*	0.00345[65]	Fresh	123†	Yes
Coleoptera	Chrysomelidae	*Oulema melanopus*	0.0061[38]		123[38]	Yes
Hemiptera	Aphididae	*Aphis gossypii*	0.000114[36]		123[36]	Yes
Coleoptera	Rutelidae	*Popillia japonica*	0.09453[55]		123[37]	Yes
Hymenoptera	Apidae	*Bombus sp.*	1.6[36]		125[36]	Yes
Hemiptera	Aphididae	*Aphis gossypii*	0.00228[66]	Fresh	125†	Yes
Hymenoptera	Vespidae	*Vespula vulgaris*	0.0841[61]		125†	Yes
Diptera	Bibionidae	*Bibio sp.*			125†	Yes
Diptera	Tephritidae	*Ceratitis capitata*	0.0049[67]		126†	Yes
Hymenoptera	Braconidae	*Cotesia glomerata*	0.001[49]		126[49]	Yes
Hemiptera	Miridae		0.0011[38]		127[38]	Yes
Hymenoptera	Apidae	*Bombus muscorum*	0.226[36]		128[36]	Yes
Hymenoptera	Apidae	*Eulaema cingulata*	0.547[36]		128[36]	No
Diptera	Drosophilidae				129†	Yes
Hymenoptera	Apidae	*Apis sp.*	0.0213[36]		130[36]	Yes
Hymenoptera	Apidae	*Bombus terrestris*	0.388[36]		130[36]	Yes
Hymenoptera	Apidae	*Xylocopa violacea*	0.614[36]		130[36]	Yes
Coleoptera	Brentidae	*Protapion fulvipes*			130†	Yes
Coleoptera	Staphylinidae	*Dalotia coriaria*			130†	Yes
Hymenoptera	Ichneumonidae	*Orthopelma mediator*	0.000712[68]	Dry	130†	Yes
Diptera	Stratiomyidae				131[42]	Yes
Diptera	Syrphidae	*Volucella bombylans*	0.1624[38]		133[38]	Yes
Diptera	Syrphidae	*Volucella pellucens*	0.01613[38]		134[38]	Yes
Diptera	Muscidae	*Musca domestica*	0.01697[69]		134†	Yes
Hymenoptera	Apidae	*Bombus liortonim*	0.159[36]		135[36]	Yes
Hymenoptera	Apidae	*Eulaema cingulata*	0.544[50]		135[50]	No
Hymenoptera	Vespidae		0.0184[38]		135[38]	Yes
Hymenoptera	Braconidae		0.0029[38]		136[38]	Yes
Diptera	Stratiomyidae				136[42]	Yes

Table continued from previous page

Order	Family	Species	Body Mass (g)	Wet/Dry/Fresh weight	WBF (Hz)	Europe (Yes/No)[35]
Diptera	Asilidae				137[42]	Yes
Diptera	Syrphidae	*Syrphus balteatus*	0.0232[36]		138[36]	Yes
Hemiptera	Miridae	*Macrolophus pygmaeus*	0.00084[70]	Fresh	139†	Yes
Hymenoptera	Vespidae	*Vespa germanica*	0.24[36]		139[36]	Yes
Hemiptera	Miridae	*Macrolophus sp.*	0.0076[38]		139[38]	Yes
Diptera	Syrphidae	*Episyrphus balteatus*	0.01507[71]		140†	Yes
Hymenoptera	Cynipidae	*Diplolepis rosae*	0.001077[68]	Dry	140†	Yes
Diptera	Cecidomyiidae	*Aphidoletes aphidimyza*			140†	Yes
Diptera	Calliphoridae	*Lucilia sericata*	0.00732[72]	Fresh	142†	Yes
Hymenoptera	Vespidae	*Vespula vulgaris*	0.09[36]		143[36]	Yes
Diptera	Syrphidae	*Chrysotoxum arcuatum*	0.073[36]		144[36]	Yes
Diptera	Psychodidae		0.0006[38]		144[38]	Yes
Hymenoptera	Apidae	*Bombus terrestris*	0.2081[38]		144[38]	Yes
Hymenoptera	Vespidae	*Vespula germanica*	0.0769[38]		145[38]	Yes
Hymenoptera	Vespidae	*Vespula germanica*	0.0833[38]		146[38]	Yes
Diptera	Syrphidae	*Platychirus pellatus*	0.0128[36]		147[36]	Yes
Diptera	Dolichopodidae	*Hydrophorus alboflorens*	0.0047[73]	Dry	148[73]	Yes
Coleoptera	Rutelidae	*Popillia japonica*	0.1955[55]		148[37]	Yes
Diptera	Tabanidae				148[42]	Yes
Hemiptera	Pentatomidae	*Graphosoma italicum*	0.045[74]	Dry	149†	Yes
Hymenoptera	Apidae	*Eulaema nigrita*	0.399[36]		149[36]	No
Hymenoptera	Apidae	*Bombus terrestris*	0.2227[38]		149[38]	Yes
Diptera	Sarcophagidae	*Sarcophaga sp.*	0.054[38]		149[38]	Yes
Hymenoptera	Apidae	*Eulaema nigrita*	0.441[50]		150[50]	No
Hymenoptera	Ichneumonidae	*Tersilochus heterocerus*			150†	Yes
Diptera	Syrphidae	*Chrysotoxum vernale*	0.064[36]		150[36]	Yes
Hemiptera	Saldidae		0.0021[75]		151†	Yes
Hemiptera	Aleyrodidae	*Trialeurodes vaporariorum*	3.5e-05[36]		151†	Yes
Diptera	Empididae		0.0193[38]		151[38]	Yes
Diptera	Sciomyzidae		0.011†	Wet	151[42]	Yes
Diptera	Tabanidae	*Haematopota pluvialis*	0.0183[38]		151[38]	Yes
Hymenoptera	Apidae	*Bombus lapidarus*	0.568[36]		152[36]	Yes
Hymenoptera	Braconidae	*Dacnusa sibirica*			152†	Yes
Hymenoptera	Vespidae	*Vespula germanica*	0.0818[38]		152[38]	Yes
Diptera	Dolichopodidae				152[42]	Yes
Hymenoptera	Braconidae	*Aphidius ervi*	0.0002[76]	Dry	152†	Yes
Hymenoptera	Apidae	*Bombus terrestris*	0.1854[38]		152[38]	Yes
Hymenoptera	Apidae	*Bombus terrestris*	0.88[36]		156[36]	Yes

Table continued from previous page

Order	Family	Species	Body Mass (g)	Wet/Dry/Fresh weight	WBF (Hz)	Europe (Yes/No)[35]
Diptera	Stratiomyidae	*Chloromyia formosa*	0.0183[38]		156[38]	Yes
Diptera	Tephritidae	*Bactrocera oleae*			157†	Yes
Diptera	Tephritidae	*Rhagoletis spp.*	0.0045†	Wet	157[42]	Yes
Hymenoptera	Braconidae	*Aphidius colemani*	3.4e-05[77]	Dry	159†	Yes
Diptera	Calliphoridae	*Calliphora sp.*	0.023[36]		160[36]	Yes
Diptera	Sarcophagidae	*Sarcophaga carnaria*	0.045[36]		160[36]	Yes
Hymenoptera	Torymidae	*Torymus sp.*	0.0024[38]		160[38]	Yes
Diptera	Calliphoridae				160[42]	Yes
Diptera	Sarcophagidae	*Sarcophaga crassipalpis*	0.02268[78]		161[79]	Yes
Diptera	Sciaridae				161[42]	Yes
Hymenoptera	Apidae	*Bombus terrestris*	0.2125[38]		161[38]	Yes
Diptera	Calliphoridae	*Calliphora erythrocephala*	0.053[36]		162[36]	Yes
Hymenoptera	Braconidae	*Aphidius matricariae*			163†	Yes
Hymenoptera			0.103[38]		163[38]	Yes
Diptera	Syrphidae	*Eristalis tenax*	0.111[36]		164[36]	Yes
Hymenoptera	Braconidae		0.003[38]		164[38]	Yes
Hymenoptera	Megachilidae	*Megachile rotundata*	0.02437[80]		165[80]	Yes
Hymenoptera	Apidae	*Bombus terrestris*	0.2154[38]		165[38]	Yes
Hemiptera	Alerodidae	*Aleurothrixus floccosus*	6.5e-05[36]		165[36]	No
Diptera	Syrphidae	*Episyrphus balteatus*	0.0294[38]		166[38]	Yes
Diptera	Drosophilidae	*Drosophila melanogaster*	0.0009[81]	Dry	166†	Yes
Diptera	Drosophilidae	*Drosophila melanogaster*	0.0006[82]	Wet	166†	Yes
Diptera	Dolichopodidae				166[42]	Yes
Diptera	Dolichopodidae				167[42]	Yes
Hymenoptera	Apidae	*Bombus terrestris*	0.1381[83]	Fresh	168†	Yes
Hymenoptera	Apidae	*Bombus terrestris*	0.1493[83]	Fresh	168†	Yes
Coleoptera	Staphylinidae	*Aleochara bilineata*	0.003[84]		168†	Yes
Hemiptera	Alerodidae	*Bemisia tabaci*	3.3e-05[36]		168[36]	Yes
Diptera	Sarcophagidae				168[42]	Yes
Diptera	Sarcophagidae	*Sarcophaga crassipalpis*	0.02268[78]		169[79]	Yes
Hymenoptera	Apidae	*Eufriesia pulchra*	0.425[36]		170[36]	No
Thysanoptera	Thripidae	*Scirtothrips citri*	1.34e-05[85]	Fresh	170†	Yes
Diptera	Lauxaniidae				170[42]	Yes
Diptera	Syrphidae	*Syrphus nitens*	0.022[36]		172[36]	Yes
Hymenoptera	Andrenidae	*Andrena sp.*	0.0453[38]		172[38]	Yes
Hymenoptera	Vespidae	*Vespula vulgaris*	0.0874[38]		173[38]	Yes
Thysanoptera	Thripidae	*Frankliniella occidentalis*	1.5e-05[86]	Fresh	174†	Yes
Diptera	Syrphidae	*Syrphus corollae*	0.0213[36]		174[36]	Yes

Table continued from previous page

Order	Family	Species	Body Mass (g)	Wet/Dry/Fresh weight	WBF (Hz)	Europe (Yes/No)[35]
Diptera	Sarcophagidae	*Sarcophaga crassipalpis*	0.02268[78]		177[79]	Yes
Diptera	Muscidae	*Mesembrina meridiana*	0.117†	Wet	177[42]	Yes
Hymenoptera	Eulophidae	*Diglyphus isaea*			177†	Yes
Diptera	Drosophilidae	*Scaptomyza flava*	0.00014[87]	Dry	177†	Yes
Diptera	Syrphidae	*Syrphus ribesii*	0.0273[38]		177[38]	Yes
Hymenoptera	Apidae	*Euglossa imperialis*	0.169[36]		179[36]	No
Diptera	Tachinidae		0.0055†	Wet	179[42]	Yes
Hemiptera	Alerodidae	*Trialeurodes vaporariorum*	3.5e-05[36]		180[36]	Yes
Diptera	Chloropidae		0.003[38]		180[38]	Yes
Hymenoptera	Apidae	*Eufriesea pulchra*	0.351[50]		181[50]	No
Hymenoptera	Apidae	*Euglossa imperialis*	0.176[50]		181[50]	No
Diptera	Calliphoridae	*Calliphora dubia*			181[79]	No
Diptera	Syrphidae	*Eristalis tenax*	0.129[36]		181[36]	Yes
Hymenoptera	Apidae	*Bombus terrestris*	0.1504[38]		183[38]	Yes
Hymenoptera	Apidae	*Euglossa cognata*	0.159[50]		184[50]	No
Diptera	Syrphidae				185[42]	Yes
Hymenoptera	Pteromalidae	*Pteromalus puparium*	0.001[49]		185[49]	Yes
Diptera	Calliphoridae				185[42]	Yes
Diptera	Heleomyzidae				185[42]	Yes
Diptera	Calliphoridae	*Calliphora dubia*			186[79]	No
Diptera	Syrphidae	*Syrphus ribesii*	0.0371[36]		186[36]	Yes
Hymenoptera	Apidae	*Bombus terrestris*	0.1641[38]		186[38]	Yes
Hymenoptera	Pteromalidae	*Spalangia cameroni*	0.025[88]	Dry	187†	Yes
Diptera	Anthomyiidae	*Delia antiqua*			187†	Yes
Diptera	Syrphidae	*Scaeva pyrastri*	0.034[36]		190[36]	Yes
Diptera	Muscidae	*Musca domestica*	0.012[36]		190[36]	Yes
Diptera	Syrphidae	*Syrphus sp.*	0.0005[38]		190[38]	Yes
Diptera	Calliphoridae	*Calliphora dubia*			191[79]	No
Hymenoptera	Megachilidae	*Osmia bicornis*	0.08345[89]	Fresh	194†	No
Diptera	Drosophilidae	*Drosophila virilis*	0.002[36]		195[36]	Yes
Diptera	Syrphidae	*Syrphus vitripennis*	0.0385[36]		196[36]	Yes
Hymenoptera	Apidae	*Apis mellifera*	0.1008[36]		197[36]	Yes
Hymenoptera	Apidae	*Bombus pascuorum*	0.1166[38]		198[38]	Yes
Diptera	Syrphidae		0.0044[38]		198[38]	Yes
Hymenoptera	Figitidae	*Leptopilina boulardi*	0.004[49]		198[49]	Yes
Diptera	Calliphoridae				199[42]	Yes
Hymenoptera	Apidae	*Bombus lapidarius*	0.1536[38]		199[38]	Yes

Table continued from previous page

Order	Family	Species	Body Mass (g)	Wet/Dry/Fresh weight	WBF (Hz)	Europe (Yes/No)[35]
Hymenoptera	Apidae	*Euglossa championi*	0.136[50]		200[50]	No
Diptera	Cecidomyiidae	*Dasineura brassicae*			200†	Yes
Diptera	Calliphoridae				200[42]	Yes
Diptera	Cecidomyiidae	*Feltiella acarisuga*			201†	Yes
Diptera	Calliphoridae	*Lucilia sericata*	0.00732[72]	Fresh	201[79]	Yes
Diptera	Chloropidae		0.00075†	Wet	202[42]	Yes
Diptera	Syrphidae				202[42]	Yes
Hymenoptera	Encyrtidae	*Leptomastix dactylopii*	0.00045[90]	Fresh	203†	No
Hymenoptera	Apidae	*Euglossa crasipunctata*	0.067[50]		204[50]	No
Hymenoptera	Apidae	*Bombus pascuorum*	0.1166[38]		205†	No
Hymenoptera	Apidae	*Euglossa bursigera*	0.084[50]		207[50]	No
Diptera	Drosophilidae				207[42]	Yes
Diptera			0.0268[38]		208[38]	Yes
Diptera	Syrphidae		0.0385[38]		208[38]	Yes
Hymenoptera	Apidae	*Euglossa mandibularis*	0.09[36]		209[36]	No
Diptera	Fanniidae	*Fannia scalaris*	0.01[36]		210[36]	Yes
Diptera	Syrphidae	*Eristalis tenax*	0.073[36]		210[36]	Yes
Hymenoptera	Crabonidae	*Ectemnius cavifrons*	0.08[38]		210[38]	Yes
Diptera	Syrphidae	*Eristalis arbustorum*	0.0705[36]		211[36]	Yes
Diptera	Syrphidae	*Eupeodes corollae*	0.01095[71]		212†	Yes
Diptera	Calliphoridae	*Lucilia sericata*	0.00768[91]	Fresh	213[79]	Yes
Hymenoptera	Andrenidae	*Andrena sp.*	0.0376[38]		213[38]	Yes
Hymenoptera	Apidae	*Euglossa tridentata*	0.11[50]		214[50]	No
Diptera	Calliphoridae	*Calliphora vomitoria*	0.0549[38]		214[38]	Yes
Diptera	Sepsidae				215[42]	Yes
Hymenoptera	Apidae	*Apis mellifera*	0.089†		217†	Yes
Diptera	Chironomidae				218[42]	Yes
Hymenoptera	Apidae	*Apis mellifera*	0.089†		219†	Yes
Diptera	Muscidae	*Hydrotaea aenescens*	0.0121[92]		219†	Yes
Hymenoptera	Vespidae	*Polistes gallicus*	0.115[36]		220[36]	Yes
Hymenoptera	Apidae	*Euglossa dissimula*	0.104[36]		220[36]	No
Diptera	Muscidae	*Musca vetustissima*	0.01[93]	Fresh	221[79]	No
Hymenoptera	Apidae	*Euglossa mixta*	0.094[50]		223[50]	No
Diptera	muscidae	*Musca vetustissima*	0.01[93]	Fresh	224[79]	No
Hemiptera	Alerodidae	*Trialeurodes abutiloides*	5e-05[36]		224[36]	Yes
Diptera	Calliphoridae	*Lucilia sericata*	0.00768[91]	Fresh	225[79]	Yes
Diptera	Sciaridae		0.00022†	Wet	225†	Yes
Hymenoptera	Apidae	*Euglossa dissimula*	0.1[50]		227[50]	No

Table continued from previous page

Order	Family	Species	Body Mass (g)	Wet/Dry/Fresh weight	WBF (Hz)	Europe (Yes/No)[35]
Hymenoptera	Apidae	*Apis mellifera*	0.089†		228†	Yes
Diptera	Muscidae	*Musca vetustissima*	0.01[93]	Fresh	229[79]	No
Diptera	Drosophilidae	*Drosophila suzukii*	0.0008[94]	Fresh	230†	Yes
Hymenoptera	Apidae	*Apis mellifera*	0.089†		230†	Yes
Hymenoptera	Apidae	*Apis mellifera*	0.0886[38]		230[38]	Yes
Hymenoptera	Apidae	*Euglossa hansoni*	0.082[50]		233[50]	No
Hymenoptera	Apidae	*Euglossa sapphirina*	0.055[50]		234[50]	No
Hymenoptera	Megachilidae	*Megachile rotundata*	0.02437[80]		234†	Yes
Hymenoptera	Apidae	*Euglossa despecta*	0.112[50]		238[50]	No
Hymenoptera	Apidae	*Apis mellifera*	0.085[36]		240[36]	Yes
Diptera	Drosophilidae	*Drosophila virilis*	0.002[36]		240[36]	Yes
Diptera	Dolichopodidae				244[42]	Yes
Hymenoptera	Apidae	*Euglossa heterosticta*	0.064[50]		250[50]	No
Hymenoptera	Apidae	*Apis mellifera*	0.078[36]		250[36]	Yes
Hymenoptera	Aphelinidae	*Encarsia formosa*			259†	Yes
Diptera	Culicidae	*Theobaldia anulata*	0.0099[36]		262[36]	Yes
Diptera	Chamaemyidae				262[42]	Yes
Hymenoptera	Apidae	*Euglossa saphirina*	0.071[36]		265[36]	No
Diptera	Pipunculidae		0.001†	Wet	269[42]	Yes
Diptera	Chloropidae	*Thaumatomyia notata*	0.0014[38]		269[38]	Yes
Hymenoptera	Trichogrammatidae	*Trichogramma*			272†	Yes
Diptera	Culicidae		0.0058[36]		277[36]	Yes
Diptera	Culicidae				286[42]	Yes
Diptera	Culicidae	*Aedes cantans*	0.0066[38]		286[38]	Yes
Diptera	Culicidae				298[42]	Yes
Diptera	Syrphidae	*Sphaerophoria scripta*	0.0193[36]		308[36]	Yes
Diptera	Culicidae		0.0018†	Wet	329†	Yes
Diptera	Culicidae	*Culiseta annulata*	0.0077[38]		331[38]	Yes
Diptera	Culicidae	*Culex pipiens*	0.0049[38]		334[38]	Yes
Diptera	Culicidae				339[42]	Yes
Diptera	Culicidae	*Culex restuans*	0.0018†	Wet	341[95]	No
Diptera	Culicidae	*Culiseta annulata*	0.007[38]		344[38]	Yes
Diptera	Culicidae	*Aedes dorsalis*	0.0018†	Wet	373[95]	Yes
Diptera	Culicidae	*Aedes vexans*	0.0024[96]	Dry	377[95]	Yes
Diptera	Culicidae	*Aedes infirmatus*	0.0018†	Wet	380[95]	Yes
Diptera	Culicidae	*Aedes japonicus*	0.0018†	Wet	383[95]	Yes
Diptera	Culicidae	*Culex coronator*	0.0018†	Wet	393[95]	No
Diptera	Culicidae	*Mansonia titillans*	0.0018†	Wet	395[95]	No

Table continued from previous page

Order	Family	Species	Body Mass (g)	Wet/Dry/Fresh weight	WBF (Hz)	Europe (Yes/No)[35]
Diptera	Culicidae	*Aedes triseriatus*	0.0005[97]	Dry	395[95]	Yes
Diptera	Culicidae	*Culex tarsalis*	0.00187[98]	Dry	395[95]	No
Diptera	Culicidae	*Culex nigripalpus*	0.0018†	Wet	397[95]	No
Diptera	Culicidae	*Psorophora columbiae*	0.0018†	Wet	411[95]	No
Diptera	Culicidae	*Anopheles crucians*	0.0018†	Wet	411[95]	No
Diptera	Culicidae	*Aedes sierrensis*	0.0018†	Wet	425[95]	No
Diptera	Culicidae	*Culiseta incidens*	0.0018†	Wet	426[95]	No
Diptera	Culicidae	*Deinocerites cancer*	0.0018†	Wet	427[95]	No
Diptera	Culicidae	*Wyeomyia mitchellii*	0.0018†	Wet	438[95]	No
Diptera	Culicidae	*Culex interrogator*	0.0018†	Wet	441[95]	No
Diptera	Chironomidae				445[42]	Yes
Diptera	Culicidae	*Aedes taeniorhynchus*	0.0018†	Wet	447[95]	No
Diptera	Culicidae	*Wyeomyia vanduzeei*	0.0018†	Wet	454[95]	No
Diptera	Culicidae	*Culex quinquefasciatus*	0.0018†	Wet	456[95]	Yes
Diptera	Culicidae	*Anopheles albimanus*	0.0018†	Wet	460[95]	No
Diptera	Culicidae	*Culex iolambdis*	0.0018†	Wet	469[95]	No
Diptera	Culicidae	*Aedes aegypti*	0.0015[36]		480[36]	Yes
Diptera	Culicidae	*Aedes aegypti*	0.0023[99]		498[95]	Yes
Diptera	Culicidae	*Anopheles quadrimaculatus*	0.0018†	Wet	504[95]	No
Diptera	Culicidae	*Wyeomyia smithii*	0.0018†	Wet	525[95]	No
Diptera	Culicidae	*Aedes albopictus*	0.0004[100]	Dry	536[95]	No
Diptera	Chironomidae		0.0018[38]		544[38]	No
Diptera	Chironomidae		0.0011[38]		557[38]	Yes
Diptera	Culicidae	*Toxorhynchites rutilus*	0.0018†	Wet	600[95]	No
Diptera	Culicidae	*Uranotaenia lowii*	0.0018†	Wet	736[95]	No
Diptera	Culicidae	*Aedes aegypti*	0.0004[99]		852†	Yes
Hemiptera	Dicytyopharidae	*Engela minuta*	0.0057[101]			No
Hemiptera	Dicytyopharidae	*Thanatodictya praeferrata*	0.0081[101]			No
Hemiptera	Dicytyopharidae	*Raphiophora vitrea*	0.0196[101]			No
Hemiptera	Dicytyopharidae	*Dictyophara europaea*	0.0229[101]			Yes
Hemiptera	Miridae	*Plagiognathus sp.*	0.0022[102]			Yes
Hemiptera	Miridae	*Orthocephalus saltator*	0.0044[102]			Yes
Hemiptera	Miridae	*Phytocoris varipes*	0.01005[102]			Yes
Hemiptera	Miridae	*Psallus perrisi*	0.0133[102]			Yes
Coleoptera	Oedemeridae	*Oedemera nobilis*	0.0189[103]			Yes
Hemiptera	Alerodidae	*Dialeurodes citri (male)*	3.6e-05[36]			Yes
Hemiptera	Alerodidae	*Dialeurodes citri (female)*	8e-05[36]			Yes

Table continued from previous page

Order	Family	Species	Body Mass (g)	Wet/Dry/Fresh weight	WBF (Hz)	Europe (Yes/No)[35]
Hymenoptera	Braconidae	*Aphidius ervi*	0.00017[104]			Yes
Coleoptera	Staphylinidae	*Aleochara lanuginosa*	0.0003[74]	Dry		No
Hemiptera	Cixiidae		0.0002[74]	Dry		Yes
Hymenoptera	Formicidae	*Lasius alienus*	0.0003[74]	Dry		Yes
Hymenoptera	Formicidae	*Ponera coarctata*	0.0003[74]	Dry		Yes
Hymenoptera	Formicidae	*Lasius niger*	0.0004[74]	Dry		Yes
Coleoptera	Carabidae	*Epaphius secalis*	0.0005[74]	Dry		Yes
Hymenoptera	Formicidae	*Lasius fuliginosus*	0.0005[74]	Dry		Yes
Coleoptera	Staphylinidae	*Oxytelus sculptus*	0.0006[74]	Dry		Yes
Hymenoptera	Formicidae	*Myrmica ruginodis*	0.0007[74]	Dry		Yes
Coleoptera	Staphylinidae	*Tachinus signatus*	0.0008[74]	Dry		Yes
Diptera	Chironomidae	*Chironomus plumosus*	0.001[74]	Dry		Yes
Coleoptera	Staphylinidae	*Philonthus rectangulus*	0.002[74]	Dry		Yes
Hemiptera	Miridae	*Lygus pratensis*	0.002[74]	Dry		Yes
Dermaptera	Spongiphoridae	*Labia minor*	0.003[74]	Dry		Yes
Hemiptera	Rhyparochromidae	*Rhyparochromus phoeniceus*	0.004[74]	Dry		Yes
Coleoptera	Histeridae	*Hister fenestus*	0.006[74]	Dry		Yes
Coleoptera	Carabidae	*Leistus ferrugineus*	0.007[74]	Dry		Yes
Coleoptera	Scarabaeidae	*Aphodius foetens*	0.009[74]	Dry		Yes
Coleoptera	Carabidae	*Ophonus rufibarbis*	0.011[74]	Dry		Yes
Coleoptera	Staphylinidae	*Philonthus decorus*	0.012[74]	Dry		Yes
Coleoptera	Carabidae	*Limodromus assimilis*	0.015[74]	Dry		Yes
Hemiptera	Coreidae	*Coreus marginatus*	0.023[74]	Dry		Yes
Coleoptera	Carabidae	*Calathus ambiguus*	0.025[74]	Dry		Yes
Coleoptera	Carabidae	*Calathus fuscipes*	0.025[74]	Dry		Yes
Coleoptera	Carabidae	*Nebria brevicollis*	0.029[74]	Dry		Yes
Dermaptera	Forficulidae	*Forficula auricularia*	0.029[74]	Dry		Yes
Hemiptera	Pyrrhocoridae	*Pyrrhocoris apterus*	0.032[74]	Dry		Yes
Coleoptera	Silphidae	*Phosphuga atrata*	0.042[74]	Dry		Yes
Coleoptera	Cetoniidae	*Oxythyrea funesta*	0.045[74]	Dry		Yes
Coleoptera	Carabidae	*Pseudoophonus rufipes*	0.047[74]	Dry		Yes
Orthoptera	Acrididae	*Chorthippus spp.*	0.063[74]	Dry		Yes
Coleoptera	Carabidae	*Pterostichus melanarius*	0.071[74]	Dry		Yes
Orthoptera	Acrididae	*Chorthippus spp.*	0.072[74]	Dry		Yes
Coleoptera	Carabidae	*Pterostichus niger*	0.074[74]	Dry		Yes
Coleoptera	Silphidae	*Silpha carinata*	0.074[74]	Dry		Yes
Coleoptera	Carabidae	*Carabus granulatus*	0.106[74]	Dry		Yes

Table continued from previous page

Order	Family	Species	Body Mass (g)	Wet/Dry/Fresh weight	WBF (Hz)	Europe (Yes/No)[35]
Coleoptera	Carabidae	*Carabus convexus*	0.12[74]	Dry		Yes
Coleoptera	Carabidae	*Carabus hortensis*	0.237[74]	Dry		Yes
Coleoptera	Carabidae	*Carabus coriaceus*	1.043[74]	Dry		Yes
Diptera	Muscidae	*Musca domestica*	0.0195[92]			Yes
Hymenoptera	Pteromalidae	*Trichilogaster signiventris*	0.00018[105]	Wet		No
Coleoptera	Chrysomelidae		0.0055[105]	Wet		Yes
Diptera	Tephritidae	*Ceratitis capitata*	0.007[105]	Wet		Yes
Hymenoptera	Pteromalidae	*Trichilogaster acaciaelongifoliae*	0.027[105]	Wet		Yes
Coleoptera	Curculionidae	*Gonipterus scutelatus*	0.03[105]	Wet		Yes
Lepidoptera	Nymphalidae	*Dira clytus*	0.062[105]	Wet		Yes
Hemiptera	Aphididae	*Aphis gossypii*	0.000183[66]	Fresh		Yes
Hymenoptera	Vespidae	*Polistes gallicus*	0.06324[106]			Yes
Hymenoptera	Vespidae	*Polistes dominula*	0.10044[106]			Yes
Coleoptera	Chrysomelidae	*Chaetocnema aridula*	0.0014[107]			Yes
Coleoptera	Chrysomelidae	*Longitarsus pratensis*	0.00175[107]			Yes
Coleoptera	Chrysomelidae	*Crepidodera aurata*	0.00265[107]			Yes
Coleoptera	Chrysomelidae	*Aphtona cyparissiae*	0.0044[107]			Yes
Coleoptera	Chrysomelidae	*Sphaeroderma testaceum*	0.00465[107]			Yes
Diptera	Tephritidae	*Ceratitis capitata*	0.00784[108]	Fresh		Yes
Diptera	Hybotidae		0.00063†	Wet		Yes
Diptera	Limoniidae		0.0018†	Wet		Yes
Diptera	Ptychopteridae	*Ptychoptera spp.*	0.0046†	Wet		Yes
Diptera	Psilidae		0.0058†	Wet		Yes
Diptera	Dolichopodidae		0.008†	Wet		Yes
Diptera	Pallopteridae					Yes
Diptera	Dolichopodidae		0.003†	Wet		

S3: Body mass and WBF distribution

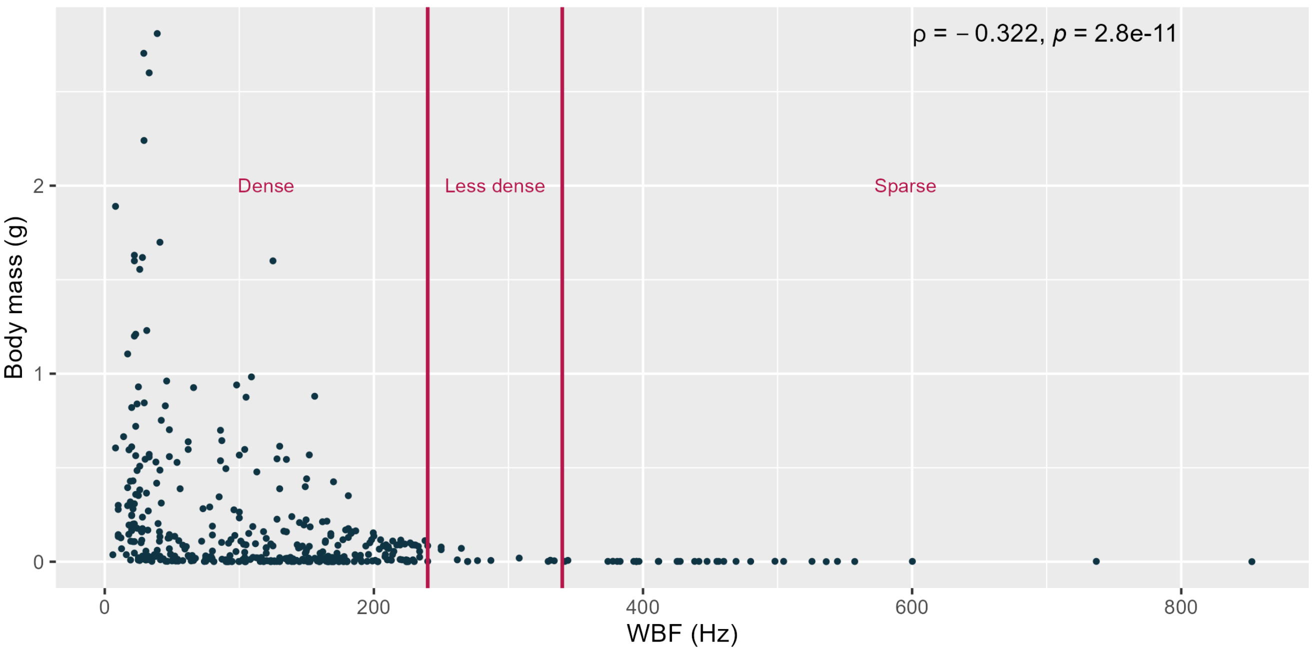

S3: Body mass and WBF distribution categorized into three regions along with Pearson's correlation coefficient ρ and its P-value. $n = 407$. From left to right: The dense region which includes WBFs less than 240 Hz. The less dense region which includes WBFs between 240 Hz and 340 Hz, and the sparse region which covers WBFs greater than 340 Hz.

S4: Biomass algorithm

Input: Set of N insect events, each with a WBF estimate μ^i_{f0}
Output: Estimated body mass B_i for each event
for $i \leftarrow 1$ to N **do**
    Retrieve candidate species from reference table
    Let C = number of retrieved candidates
    if $C = 0$ **then**
        if $240 < \mu^i_{f_0} \leq 340$ Hz **then**
            $B_i \leftarrow$ median mass for $(240 < \mu^i_{f_0} \leq 340$ Hz$)$
        else if $\mu^i_{f_0} > 340$ Hz **then**
            $B_i \leftarrow$ mass for $\mu^i_{f_0} = 480$ Hz (mosquito)
        else
            $B_i \leftarrow$ median mass for $\mu^i_{f_0} \leq 240$ Hz
        end if
    else if $C = 1$ **then**
        $B_i \leftarrow$ mass of the single candidate
    else $\triangleright C > 1$
        Exclude non-region insects (e.g., non-European) to refine the candidate set
        Let C' = updated number of candidates
        if $C' = 0$ **then**
            $B_i \leftarrow 0$
        else if $C' = 1$ **then**
            $B_i \leftarrow$ mass of the single remaining candidate
        else
            $B_i \leftarrow$ weighted average of remaining candidate masses
        end if
    end if
end for

S5: Descriptive statistics of reference table

Descriptive statistics for body mass and WBF from the reference table (S2), including number of values (N), mean, standard deviation (S.D.), minimum (Min), and maximum (Max) values.

Variable	N	Mean	S.D.	Min	Max
Body Mass (g)	480	0.17	0.36	0.000013	2.8
WBF (Hz)	473	143	117	6	852

S6: Overview of count and biomass samples

Start- and end date of count and biomass data collection for both sites 1 and 2 for both sensors and Malaise traps. The table is ordered by site and start date.

Start Date	End Date	Site	Sensor Count	Malaise Count	Sensor Biomass (g)	Malaise Biomass (g)
2024-06-17	2024-06-18	Site1	329	320	25.0	1.645
2024-06-18	2024-06-19	Site1	221	385	14.141136	1.593
2024-06-19	2024-06-20	Site1	97	103	12.674007	0.43
2024-06-20	2024-06-21	Site1	176	482	13.991867	1.368
2024-06-24	2024-06-25	Site1	503	463	16.8197	1.451
2024-06-25	2024-06-26	Site1	241	797	19.701242	2.16
2024-06-26	2024-06-27	Site1	313	380	31.594295	1.86
2024-06-27	2024-06-28	Site1	301	832	30.040947	3.271
2024-07-11	2024-07-15	Site1	2033	1091	113.545189	4.322
2024-07-15	2024-07-18	Site1	3602	916	195.0	3.033
2024-07-18	2024-07-22	Site1	8856	2799	350.0	10.757
2024-07-22	2024-07-25	Site1	2896	892	156.0	5.142
2024-07-25	2024-07-29	Site1	3243	996	163.0	4.69
2024-07-29	2024-08-01	Site1	6010	1560	205.0	5.122
2024-08-01	2024-08-05	Site1	7331	1498	237.33	5.65
2024-08-05	2024-08-08	Site1	8700	969	296.39	3.782
2024-08-08	2024-08-12	Site1	2582	682	152.13	2.073
2024-08-12	2024-08-15	Site1	4948	779	202.53	2.651
2024-08-15	2024-08-19	Site1	1006	1003	71.5	2.25
2024-08-19	2024-08-22	Site1	1442	426	88.13	0.848
2024-08-22	2024-08-26	Site1	2482	436	297.35	1.005
2024-08-26	2024-08-29	Site1	1081	650	145.36	4.683
2024-08-29	2024-09-02	Site1	1084	616	121.17	2.121
2024-09-02	2024-09-05	Site1	1055	547	118.404	1.82
2024-09-05	2024-09-09	Site1	747	716	95.13	1.454
2024-09-09	2024-09-12	Site1	103	137	28.23	0.801
2024-06-17	2024-06-18	Site2	944	948	51.311	4.38
2024-06-18	2024-06-19	Site2	1048	921	23.9105605	3.827
2024-06-19	2024-06-20	Site2	207	322	45.44451767	0.964
2024-06-20	2024-06-21	Site2	2361	1162	71.00112367	2.804
2024-06-24	2024-06-25	Site2	881	621	36.41	2.161
2024-06-25	2024-06-26	Site2	901	1071	33.29	3.067
2024-06-26	2024-06-27	Site2	864	1097	49.42	3.237

Continue on next page

Table continued from previous page

Start Date	End Date	Location	Sensor Count	Malaise Count	Sensor Biomass (g)	Malaise Biomass (g)
2024-06-27	2024-06-28	Site2	1692	1551	34.56	5.437
2024-07-11	2024-07-15	Site2	1990	1702	152.41	3.111
2024-07-15	2024-07-18	Site2	1154	587	281.87	2.373
2024-07-18	2024-07-22	Site2	4735	2995	378.22	11.275
2024-07-22	2024-07-25	Site2	3574	1538	362.82	4.074
2024-07-25	2024-07-29	Site2	9897	2023	621.45	6.865
2024-07-29	2024-08-01	Site2	11343	733	425.06	2.811
2024-08-01	2024-08-05	Site2	15317	3057	601.0	8.162
2024-08-05	2024-08-08	Site2	10782	1745	519.63	5.97
2024-08-08	2024-08-12	Site2	2575	2115	393.24	4.762
2024-08-12	2024-08-15	Site2	2313	2095	340.06	6.45
2024-08-15	2024-08-19	Site2	1223	2476	291.71	4.313
2024-08-19	2024-08-22	Site2	982	973	256.96	2.238
2024-08-22	2024-08-26	Site2	2033	782	710.89	1.859
2024-08-26	2024-08-29	Site2	3034	1224	553.76	4.179
2024-08-29	2024-09-02	Site2	3529	1610	253.68	12.3
2024-09-02	2024-09-05	Site2	2777	845	111.39	7.11
2024-09-05	2024-09-09	Site2	2301	604	68.71	2.213
2024-09-09	2024-09-12	Site2	629	171	89.23	1.014

Concluded

S7: Descriptive statistics of count and biomass values for site 1

Descriptive statistics for count and biomass for site 1 from the sample overview table (S4), including number of values (N), mean, standard deviation (S.D.), minimum (Min), and maximum (Max) values.

Variable	N	Mean	S.D.	Min	Max
Sensor Count	26	2361	2685	97	8856
Malaise Count	26	788	544	103	2799
Sensor Biomass (g)	26	123	99	13	350
Malaise Biomass (g)	26	2.9	2.2	0.43	11

S8: Descriptive statistics of count and biomass values for site 2

Descriptive statistics for count and biomass for site 2 from the sample overview table (S4), including number of values (N), mean, standard deviation (S.D.), minimum (Min), and maximum (Max) values.

Variable	N	Mean	S.D.	Min	Max
Sensor Count	26	3426	3894	207	15317
Malaise Count	26	1345	766	171	3057
Sensor Biomass (g)	26	260	215	24	711
Malaise Biomass (g)	26	4.5	2.9	0.96	12

S9: Shapiro-Wilk's test for normality

Shapiro-Wilk's test results for all variables for both sites 1-2, including P-value and level of significance:

$P < 0.001$ ***
$P < 0.01$ **
$P < 0.05$ *
$P > 0.05$ NS

Variable	P-Value	Significance
Site 1 - Malaise Count	0.000341	***
Site 1 - Malaise Biomass	0.000429	***
Site 1 - Sensor Count	0.000133	***
Site 1 - Sensor Biomass	0.024360	*
Site 2 - Malaise Count	0.156467	NS
Site 2 - Malaise Biomass	0.006753	**
Site 2 - Sensor Count	0.000006	***
Site 2 - Sensor Biomass	0.010702	*

References

35. GBIF.org. GBIF home page. https://www.gbif.org (2025). Accessed: 28 January 2025.

36. Byrne, D. N., Buchmann, S. L. & Spangler, H. G. Relationship Between Wing Loading, Wingbeat Frequency and Body Mass in Homopterous Insects. *J. Exp. Biol.* **135**, 9–23, DOI: https://doi.org/10.1242/JEB.135.1.9 (1988).

37. Yu, W., Zhang, H., Xu, R., Sun, Y. & Wu, K. Characterization of Wingbeat Frequency of Different Taxa of Migratory Insects in Northeast Asia. *Insects* **13**, 520, DOI: https://doi.org/10.3390/INSECTS13060520 (2022).

38. Tercel, M. P., Veronesi, F. & Pope, T. W. Phylogenetic clustering of wingbeat frequency and flight-associated morphometrics across insect orders. *Physiol. Entomol.* **43**, 149–157, DOI: https://doi.org/10.1111/PHEN.12240 (2018).

39. Burrows, M. & Dorosenko, M. Jumping mechanisms in lacewings (Neuroptera, Chrysopidae and Hemerobiidae). *J. Exp. Biol.* **217**, 4252–4261, DOI: https://doi.org/10.1242/JEB.110841 (2014).

40. Sarfraz, M., Dosdall, L. M. & Keddie, B. A. Resistance of Some Cultivated Brassicaceae to Infestations by Plutella xylostella (Lepidoptera: Plutellidae). *J. Econ. Entomol.* **100**, 215–224, DOI: https://doi.org/10.1093/JEE/100.1.215 (2007).

41. Ankersmit, G. W., Rabbinge, R. & Dijkman, H. Studies on the sterile-male technique as a means of control of adoxophyes orana (Lepidoptera, Tortricidae) 4. technical and economic aspects of mass-rearing. *Neth. J. Plant Pathol.* **83**, 27–39, DOI: https://doi.org/10.1007/BF01976509 (1977).

42. Hall, J. M. *et al.* Kinematic diversity suggests expanded roles for fly halteres. *Biol. Lett.* **11**, 20150845, DOI: https://doi.org/10.1098/RSBL.2015.0845 (2015).

43. Sambaraju, K. R. & Phillips, T. W. Ovipositional preferences and larval performances of two populations of indianmeal moth, Plodia interpunctella. *Entomol. Exp. et Appl.* **128**, 283–293, DOI: https://doi.org/10.1111/J.1570-7458.2008.00723.X (2008).

44. Li, Z., Li, D., Xie, B., Ji, R. & Cui, J. Effect of body size and larval experience on mate preference in Helicoverpa armigera (Hübner) (Lep., Noctuidae). *J. Appl. Entomol.* **129**, 574–579, DOI: https://doi.org/10.1111/J.1439-0418.2005.01012.X (2005).

45. Gu, H., Hughes, J. & Dorn, S. Trade-off between mobility and fitness in Cydia pomonella L. (Lepidoptera: Tortricidae). *Ecol. Entomol.* **31**, 68–74, DOI: https://doi.org/10.1111/J.0307-6946.2006.00761.X (2006).

46. Grabow, K. & Rüppell, G. Wing loading in relation to size and flight characteristics of European Odonata. *Odonatologica* **24**, 175–186 (1995).

47. Torres-Vila, L. M., Rodríguez-Molina, M. C., Roehrich, R. & Stockel, J. Vine phenological stage during larval feeding affects male and female reproductive output of Lobesia botrana (Lepidoptera: Tortricidae). *Bull. Entomol. Res.* **89**, 549–556, DOI: https://doi.org/10.1017/S000748539900070X (1999).

48. Lehmann, P., Westberg, M., Tang, P., Lindström, L. & Käkelä, R. The Diapause Lipidomes of Three Closely Related Beetle Species Reveal Mechanisms for Tolerating Energetic and Cold Stress in High-Latitude Seasonal Environments. *Front. Physiol.* **11**, 576617, DOI: https://doi.org/10.3389/fphys.2020.576617 (2020).

49. Burrows, M. & Dorosenko, M. Take-off mechanisms in parasitoid wasps. *J. Exp. Biol.* **220**, 3812–3825, DOI: https://doi.org/10.1242/JEB.161463 (2017).

50. Darveau, C. A., Hochachka, P. W., Welch, K. C., Roubik, D. W. & Suarez, R. K. Allometric scaling of flight energetics in Panamanian orchid bees: a comparative phylogenetic approach. *J. Exp. Biol.* **208**, 3581–3591, DOI: https://doi.org/10.1242/JEB.01776 (2005).

51. Müller, C. Interactions between glucosinolate- and myrosinase-containing plants and the sawfly Athalia rosae. *Phytochem. Rev.* **8**, 121–134, DOI: https://doi.org/10.1007/s11101-008-9115-3 (2009).

52. Hondelmann, P., Paul, C., Schreiner, M. & Meyhöfer, R. Importance of Antixenosis and Antibiosis Resistance to the Cabbage Whitefly (Aleyrodes proletella) in Brussels Sprout Cultivars. *Insects* **11**, 56, DOI: https://doi.org/10.3390/INSECTS11010056 (2020).

53. Acebes-Doria, A. L., Leskey, T. C. & Bergh, J. C. Host Plant Effects on Halyomorpha halys (Hemiptera: Pentatomidae) Nymphal Development and Survivorship. *Environ. Entomol.* **45**, 663–670, DOI: https://doi.org/10.1093/EE/NVW018 (2016).

54. Raworth, D. A., McFarlane, S., Gilbert, N. & Frazer, B. D. Population dynamics of the cabbage aphid, Brevicoryne brassicae (Homoptera: Aphididae) at Vancouver, British Columbia: III. development, fecundity, and morph determination vs. aphid density and plant quality. *The Can. Entomol.* **116**, 879–888, DOI: https://doi.org/10.4039/ENT116879-6 (1984).

55. Oertli, J. J. & Oertli, M. Energetics and thermoregulation of Popillia japonica Newman (Scarabaeidae, Coleoptera) during flight and rest. *Physiol. Zool.* **63**, DOI: https://doi.org/10.1086/physzool.63.5.30152621 (1990).

56. Dixon, A. F. G., Chambers, R. J. & Dharma, T. R. Factors affecting size in aphids with particular reference to the black bean aphid, Aphis fabae. *Entomol. Exp. et Appl.* **32**, 123–128, DOI: https://doi.org/10.1111/j.1570-7458.1982.tb03192.x (1982).

57. Kempton, R. A., Lowe, H. J. B. & Bintcliffe, E. J. B. The Relationship between Fecundity and Adult Weight in Myzus persicae. *The J. Animal Ecol.* **49**, 917, DOI: https://doi.org/10.2307/4235 (1980).

58. Käfer, H., Kovac, H., Oswald, B. & Stabentheiner, A. Respiration and metabolism of the resting european paper wasp (Polistes dominulus). *J. Comp. Physiol. B: Biochem. Syst. Environ. Physiol.* **185**, 647–658, DOI: https://doi.org/10.1007/S00360-015-0915-7 (2015).

59. Ludwig, D. The Effect of Temperature on the Growth Curves of the Japanese Beetle (Popillia japonica Newman). *Physiol. Zool.* **5**, DOI: https://doi.org/10.1086/physzool.5.3.30151174 (1932).

60. Giovanetti, M. & Lasso, E. Body size, loading capacity and rate of reproduction in the communal bee Andrena agilissima (Hymenoptera; Andrenidae). *Apidologie* **36**, 439–447, DOI: https://doi.org/10.1051/APIDO:2005028 (2005).

61. Kovac, H. & Stabentheiner, A. Does size matter? – Thermoregulation of 'heavyweight' and 'lightweight' wasps (Vespa crabro and Vespula sp.). *Biol. Open* **1**, 848–856, DOI: https://doi.org/10.1242/BIO.20121156 (2012).

62. Willow, J. *et al.* Evaluating the effect of seven plant essential oils on pollen beetle (Brassicogethes aeneus) survival and mobility. *Crop. Prot.* **134**, 105181, DOI: https://doi.org/10.1016/J.CROPRO.2020.105181 (2020).

63. Grayson, F. W. L. & Hassall, M. Effects of Rabbit Grazing on Population Variables of Chorthippus brunneus (Orthoptera). *Oikos* **44**, 27, DOI: https://doi.org/10.2307/3544039 (1985).

64. Groot, A. T. & Smid, H. M. Polyandry in the mind bug Lygocoris pabulinus (L.)—effects on sexual communication and fecundity. *Invertebr. Reproduction & Dev.* **38**, 143–155, DOI: https://doi.org/10.1080/07924259.2000.9652449 (2000).

65. Chuche, J. & Thiéry, D. Egg incubation temperature differently affects female and male hatching dynamics and larval fitness in a leafhopper. *Ecol. Evol.* **2**, DOI: https://doi.org/10.1002/ece3.89 (2012).

66. Jiang, S. *et al.* Feeding behavioral response of cotton aphid, Aphis gossypii, to elevated CO2: EPG test with leaf microstructure and leaf chemistry. *Entomol. Exp. et Appl.* **160**, 219–228, DOI: https://doi.org/10.1111/EEA.12475 (2016).

67. Esterhuizen, N. *et al.* Effects of within-generation thermal history on the flight performance of Ceratitis capitata: Colder is better. *J. Exp. Biol.* **217**, 3545–3556, DOI: https://doi.org/10.1242/jeb.106526/257777/am/effects-of-within-generation-thermal-history-on (2014).

68. Visser, B., Dooremalen, C. V., Ruiz, A. V. & Ellers, J. Fatty acid composition remains stable across trophic levels in a gall wasp community. *Physiol. Entomol.* **38**, 306–312, DOI: https://doi.org/10.1111/PHEN.12035 (2013).

69. Neupane, S., White, K., Thomson, J. L., Zurek, L. & Nayduch, D. Environmental and Sex Effects on Bacterial Carriage by Adult House Flies (Musca domestica L.). *Insects* **11**, 401, DOI: https://doi.org/10.3390/INSECTS11070401 (2020).

70. Oliveira, L. . . *et al.* Contrasting Phenotypic Variability of Life-History Traits of Two Feral Populations of Macrolophus pygmaeus (Hemiptera: Miridae) under Two Alternative Diets. *Agronomy* **13**, 118, DOI: https://doi.org/10.3390/AGRONOMY13010118 (2022).

71. Putra, N. S. & Yasuda, H. Effects of prey species and its density on larval performance of two species of hoverfly larvae, Episyrphus balteatus de Geer and Eupeodes corollae Fabricius (Diptera: Syrphidae). *Appl. Entomol. Zool.* **41**, 389–397, DOI: https://doi.org/10.1303/AEZ.2006.389 (2006).

72. Kökdener, M. & Yllmaz, A. F. The Effects of Gunshot Residue Components (Pb, Ba, and Sb) on the Life History Traits of Lucilia sericata (Diptera: Calliphoridae). *J. Med. Entomol.* **58**, 2130–2137, DOI: https://doi.org/10.1093/JME/TJAB123 (2021).

73. Burrows, M. Jumping from the surface of water by the long-legged fly Hydrophorus (Diptera, Dolichopodidae). *J. Exp. Biol.* **216**, 1973–1981, DOI: https://doi.org/10.1242/JEB.083683 (2013).

74. Faly, L. I., Brygadyrenko, V. V., Orzekauskaite, A. & Paulauskas, A. Sensitivity of non-target groups of invertebrates to cypermethrin. *Biosyst. Divers.* **31**, 393–400, DOI: https://doi.org/10.15421/012347 (2023).

75. Burrows, M. Jumping strategies and performance in shore bugs (Hemiptera, Heteroptera,Saldidae). *J. Exp. Biol.* **212**, 106–115, DOI: https://doi.org/10.1242/JEB.024448 (2009).

76. Sequeira, R. & Mackauer, M. Covariance of adult size and development time in the parasitoid wasp Aphidius ervi in relation to the size of its host, Acyrthosiphon pisum. *Evol. Ecol.* **6**, 34–44, DOI: https://doi.org/10.1007/BF02285332/METRICS (1992).

77. Ode, P. J. & Crompton, D. S. Compatibility of aphid resistance in soybean and biological control by the parasitoid Aphidius colemani (Hymenoptera: Braconidae). *Biol. Control.* **64**, 255–262, DOI: https://doi.org/10.1016/J.BIOCONTROL.2012.12.001 (2013).

78. Bulut, M., Ünal Zeybekoğlu & Kökdener, M. Effects of Tissue Type and Temperature on Selected Life-History Traits of the Flesh Fly, Sarcophaga crassipalpis (Macquart, 1839) (Diptera: Sarcophagidae). *J. Med. Entomol.* **59**, 1921–1927, DOI: https://doi.org/10.1093/JME/TJAC149 (2022).

79. Pinto, J., Magni, P. A., O'Brien, R. C. & Dadour, I. R. Chasing Flies: The Use of Wingbeat Frequency as a Communication Cue in Calyptrate Flies (Diptera: Calyptratae). *Insects* **13**, 822, DOI: https://doi.org/10.3390/INSECTS13090822 (2022).

80. Grula, C. C., Rinehart, J. P., Greenlee, K. J. & Bowsher, J. H. Body size allometry impacts flight-related morphology and metabolic rates in the solitary bee Megachile rotundata. *J. Insect Physiol.* **133**, 104275, DOI: https://doi.org/10.1016/J.JINSPHYS.2021.104275 (2021).

81. Voorhies, W. A. V., Khazaeli, A. A. & Curtsinger, J. W. Lack of correlation between body mass and metabolic rate in Drosophila melanogaster. *J. Insect Physiol.* **50**, 445–453, DOI: https://doi.org/10.1016/J.JINSPHYS.2004.03.002 (2004).

82. Schilder, R. J. & Raynor, M. Molecular plasticity and functional enhancements of leg muscles in response to hypergravity in the fruit fly Drosophila melanogaster. *J. Exp. Biol.* **220**, 3508–3518, DOI: https://doi.org/10.1242/JEB.160523 (2017).

83. Spaethe, J. & Weidenmüller, A. Size variation and foraging rate in bumblebees (Bombus terrestris). *Insectes Sociaux* **49**, 142–146, DOI: https://doi.org/10.1007/S00040-002-8293-Z/METRICS (2002).

84. Green, K. Age-related variation in mean sperm length, in the rove beetle Aleochara bilineata. *J. Insect Physiol.* **49**, 993–998, DOI: https://doi.org/10.1016/S0022-1910(03)00164-1 (2003).

85. Wiesenborn, W. D. & Morse, J. G. Feeding rate of Scirtothrips citri (Moulton) (Thysanoptera: Thripidae) as Influenced by Life Stage and Temperature. *Environ. Entomol.* **15**, 763–766, DOI: https://doi.org/10.1093/EE/15.3.763 (1986).

86. Terry, L. I. & Dyreson, E. Behavior of Frankliniella occidentalis (Thysanoptera: Thripidae) within Aggregations, and Morphometric correlates of Fighting. *Annals Entomol. Soc. Am.* **89**, 589–602, DOI: https://doi.org/10.1093/AESA/89.4.589 (1996).

87. Whiteman, N. K. *et al.* Mining the plant–herbivore interface with a leafmining Drosophila of Arabidopsis. *Mol. Ecol.* **20**, 995–1014, DOI: https://doi.org/10.1111/J.1365-294X.2010.04901.X (2011).

88. King, B. H. Sex-ratio manipulation in response to host size by the parasitoid wasp Spalangia cameroni: a laboratory study. *Evolution* **42**, DOI: https://doi.org/10.1111/j.1558-5646.1988.tb04179.x (1988).

89. Seidelmann, K. Optimal resource allocation, maternal investment, and body size in a solitary bee, Osmia bicornis. *Entomol. Exp. et Appl.* **166**, 790–799, DOI: https://doi.org/10.1111/EEA.12711 (2018).

90. Dillon, N., Austin, A. D. & Bartowsky, E. Comparison of preservation techniques for DNA extraction from hymenopterous insects. *Insect Mol. Biol.* **5**, 21–24, DOI: https://doi.org/10.1111/J.1365-2583.1996.TB00036.X (1996).

91. Kökdener, M., Gündüz, N. E. A., Ünal Zeybekoğlu, Aykut, U. & Yllmaz, A. F. The Effect of Different Heavy Metals on the Development of Lucilia sericata (Diptera: Calliphoridae). *J. Med. Entomol.* **59**, 1928–1935, DOI: https://doi.org/10.1093/JME/TJAC134 (2022).

92. Farkas, R. & Pap, L. Susceptibility of Hydrotaea aenescens (Wiedemann) (Diptera: Muscidae) to selected insecticides and its importance in integrated fly management. *Parasitol. Res. 1996 82:2* **82**, 170–173, DOI: https://doi.org/10.1007/S004360050089 (1996).

93. Greenham, P. M. The Effects of the Variability of Cattle Dung on the Multiplication of the Bushfly (Musca vetustissima Walk.). *The J. Animal Ecol.* **41**, 153, DOI: https://doi.org/10.2307/3510 (1972).

94. Shu, R., Uy, L. & Wong, A. C. N. Nutritional phenotype underlines the performance trade-offs of Drosophila suzukii on different fruit diets. *Curr. Res. Insect Sci.* **2**, 100026, DOI: https://doi.org/10.1016/J.CRIS.2021.100026 (2022).

95. Kim, D., DeBriere, T. J., Cherukumalli, S., White, G. S. & Burkett-Cadena, N. D. Infrared light sensors permit rapid recording of wingbeat frequency and bioacoustic species identification of mosquitoes. *Sci. Reports* **11**, 1–9, DOI: https://doi.org/10.1038/s41598-021-89644-z (2021).

96. Brust, R. A. Weight and development time of different stadia of mosquitoes reared at various constant temperatures. *The Can. Entomol.* **99**, 986–993, DOI: https://doi.org/10.4039/ENT99986-9 (1967).

97. Juliano, S. A. & Stoffregen, T. L. Effects of habitat drying on size at and time to metamorphosis in the tree hole mosquito Aedes triseriatus. *Oecologia 1994 97:3* **97**, 369–376, DOI: https://doi.org/10.1007/BF00317327 (1994).

98. Gray, E. M. & Bradley, T. J. Metabolic Rate in Female Culex tarsalis (Diptera: Culicidae) : Age, Size, Activity, and Feeding Effects. *J. Med. Entomol.* **40**, 903–911, DOI: https://doi.org/10.1603/0022-2585-40.6.903 (2003).

99. Arrivillaga, J. & Barrera, R. Food as a limiting factor for Aedes aegypti in water-storage containers. *J. vector ecology : journal Soc. for Vector Ecol.* **29** (2004).

100. Reiskind, M. H. & Zarrabi, A. A. Is bigger really bigger? differential responses to temperature in measures of body size of the mosquito, Aedes albopictus. *J. Insect Physiol.* **58**, 911–917, DOI: https://doi.org/10.1016/J.JINSPHYS.2012.04.006 (2012).

101. Burrows, M. Jumping mechanisms in dictyopharid planthoppers (Hemiptera, Dicytyopharidae). *J. Exp. Biol.* **217**, 402–413, DOI: https://doi.org/10.1242/jeb.093476 (2014).

102. Burrows, M. & Dorosenko, M. Jumping performance of flea hoppers and other mirid bugs (Hemiptera, Miridae). *J. Exp. Biol.* **220**, 1606–1617, DOI: https://doi.org/10.1242/JEB.154153 (2017).

103. Burrows, M. Do the enlarged hind legs of male thick-legged flower beetles contribute to take-off or mating? *J. Exp. Biol.* **223**, DOI: https://doi.org/10.1242/JEB.212670 (2020).

104. Couty, A. *et al.* Effects of artificial diet containing GNA and GNA-expressing potatoes on the development of the aphid parasitoid Aphidius ervi Haliday (Hymenoptera: Aphidiidae). *J. Insect Physiol.* **47**, 1357–1366, DOI: https://doi.org/10.1016/S0022-1910(01)00111-1 (2001).

105. Gouws, E. J., Gaston, K. J. & Chown, S. L. Intraspecific Body Size Frequency Distributions of Insects. *PLOS ONE* **6**, e16606, DOI: https://doi.org/10.1371/JOURNAL.PONE.0016606 (2011).

106. Kovac, H., Kundegraber, B., Käfer, H., Petrocelli, I. & Stabentheiner, A. Relation between activity, endothermic performance and respiratory metabolism in two paper wasps: Polistes dominula and Polistes gallicus. *Comp. Biochem. Physiol. Part A: Mol. & Integr. Physiol.* **250**, 110804, DOI: https://doi.org/10.1016/J.CBPA.2020.110804 (2020).

107. Nadein, K. & Betz, O. Jumping mechanisms and performance in beetles. I. Flea beetles (Coleoptera: Chrysomelidae: Alticini). *J. Exp. Biol.* **219**, 2015–2027, DOI: https://doi.org/10.1242/JEB.140533 (2016).

108. Weldon, C. W., Nyamukondiwa, C., Karsten, M., Chown, S. L. & Terblanche, J. S. Geographic variation and plasticity in climate stress resistance among southern African populations of Ceratitis capitata (Wiedemann) (Diptera: Tephritidae). *Sci. Reports* **8**, 1–13, DOI: https://doi.org/10.1038/s41598-018-28259-3 (2018).
